# The effect of exposure to long working hours on alcohol consumption, risky drinking and alcohol use disorder: A systematic review and meta-analysis from the WHO/ILO Joint Estimates of the Work-related burden of disease and injury

**DOI:** 10.1016/j.envint.2020.106205

**Published:** 2021-01

**Authors:** Daniela V. Pachito, Frank Pega, Jelena Bakusic, Emma Boonen, Els Clays, Alexis Descatha, Ellen Delvaux, Dirk De Bacquer, Karoliina Koskenvuo, Hannes Kröger, Marie-Claire Lambrechts, Carolina O.C. Latorraca, Jian Li, Ana L. Cabrera Martimbianco, Rachel Riera, Reiner Rugulies, Grace Sembajwe, Johannes Siegrist, Lauri Sillanmäki, Markku Sumanen, Sakari Suominen, Yuka Ujita, Godelieve Vandersmissen, Lode Godderis

**Affiliations:** a Núcleo de Avaliação de Tecnologias em Saúde, Hospital Sírio-Libanês, Rua Barata Ribeiro 142, Bela Vista, São Paulo, Brazil; b Fundação Getúlio Vargas, Av. Paulista, 548, Bela Vista, São Paulo, Brazil; c Environment, Climate Change and Health Department, World Health Organization, 20 Avenue Appia, 1211 Geneva 27, Switzerland; d Centre for Environment and Health of KU Leuven, Kapucijnenvoer 35/5, box 7001, 3000 Leuven, Belgium; e KIR Department (Knowledge, Information & Research), IDEWE, External Service for Prevention and Protection at Work, Interleuvenlaan 58, 3001 Leuven, Belgium; f Department of Public Health and Primary Care, Ghent University, Campus University Hospital Ghent, Cornel Heymanslaan 10, B-9000 Ghent, Belgium; g AP-HP (Paris Hospital), Occupational Health Unit, Poincaré University Hospital, Garches, France; h Inserm Versailles St-Quentin Univ – Paris Saclay Univ (UVSQ), UMS 011, UMR-S 1168, Villejuif, France; i Univ Angers, CHU Angers, Univ Rennes, Inserm, EHESP, Irset (Institut de recherche en santé, environnement et travail) - UMR_S 1085, F-49000 Angers, France; j Centre for Social and Cultural Psychology of KU Leuven, Dekenstraat 2, box 3701, 3000 Leuven, Belgium; k The Social Insurance Institution of Finland, PO Box 450, FIN-00056 Kela, Finland; l Department of Public Health, PO BOX 20, 00014 University of Helsinki, Finland; m Socio-Economic Panel (SOEP), German Institute for Economic Research (DIW), Berlin, Germany; n VAD, Flemish Expertise Centre for Alcohol and Other Drugs, Vanderlindenstraat 15, Brussels, Belgium; o Discipline of Evidence-based Medicine, Universidade Federal de São Paulo, Rua Botucatu 740, Sao Paulo, Brazil; p Department of Environmental Health Sciences, Fielding School of Public Health, School of Nursing, University of California, Los Angeles, United States; q Postgraduate Program in Health and Environment, Universidade Metropolitana de Santos (UNIMES), 536 Conselheiro Nébias, Santos, Brazil; r Cochrane Brazil, Affiliate Center Rio de Janeiro, 136 Barão do Rio Branco, Petrópolis, Brazil; s Centro Universitário São Camilo, 1501 Nazaré, Sao Paulo, Brazil; t Oxford-Brazil EBM-Alliance, Brazil; u National Research Centre for the Working Environment, Lersø Parkallé 105, DK-2100 Copenhagen, Denmark; v Department of Public Health, University of Copenhagen, Øster Farimagsgade 5, DK-1014 Copenhagen, Denmark; w Department of Psychology, University of Copenhagen, Øster Farimagsgade 2A, DK-1353 Copenhagen, Denmark; x Department of Occupational Medicine, Epidemiology and Prevention (OMEP), Donald and Barbara Zucker School of Medicine at Hofstra University, 175 Community Drive, NY 11021, United States; y CUNY Institute for Implementation Science, CUNY Graduate School of Public Health and Health Policy, 55 W 125th Street, New York, NY 10027, United States; z Life Science Centre, University of Düsseldorf, Merowingerplatz 1a, D-40225 Duesseldorf, Germany; aa Department of Public Health, University of Helsinki, Mannerheimintie 172, 00300 Helsinki, Finland; ab Department of Public Health, University of Turku, Joukahaisenkatu 3-5, 20520 Turku, Finland; ac Turku Clinical Research Centre, Turku University Hospital, Finland; ad Faculty of Medicine and Health Technology, Tampere University, Tampere, Finland; ae University of Skövde, School of Health Sciences, Sweden; af Labour Administration, Labour Inspection and Occupational Safety and Health Branch, International Labour Organization, Route des Morillons 4, 1211 Geneva, Switzerland

**Keywords:** Global burden of disease, Occupational health, Long working hours, Alcohol consumption, Systematic review, Meta-analysis

## Abstract

**Background:**

The World Health Organization (WHO) and the International Labour Organization (ILO) are developing Joint Estimates of the work-related burden of disease and injury (WHO/ILO Joint Estimates), with contributions from a large network of experts. Evidence from mechanistic data suggests that exposure to long working hours may increase alcohol consumption and cause alcohol use disorder. In this paper, we present a systematic review and meta-analysis of parameters for estimating the number of deaths and disability-adjusted life years from alcohol consumption and alcohol use disorder that are attributable to exposure to long working hours, for the development of the WHO/ILO Joint Estimates.

**Objectives:**

We aimed to systematically review and meta-analyse estimates of the effect of exposure to long working hours (three categories: 41–48, 49–54 and ≥55 h/week), compared with exposure to standard working hours (35–40 h/week), on alcohol consumption, risky drinking (three outcomes: prevalence, incidence and mortality) and alcohol use disorder (three outcomes: prevalence, incidence and mortality).

**Data sources:**

We developed and published a protocol, applying the Navigation Guide as an organizing systematic review framework where feasible. We searched electronic bibliographic databases for potentially relevant records from published and unpublished studies, including the WHO International Clinical Trials Register, Ovid MEDLINE, PubMed, Embase, and CISDOC on 30 June 2018. Searches on PubMed were updated on 18 April 2020. We also searched electronic grey literature databases, Internet search engines and organizational websites; hand-searched reference list of previous systematic reviews and included study records; and consulted additional experts.

**Study eligibility and criteria:**

We included working-age (≥15 years) workers in the formal and informal economy in any WHO and/or ILO Member State but excluded children (<15 years) and unpaid domestic workers. We considered for inclusion randomized controlled trials, cohort studies, case-control studies and other non-randomized intervention studies with an estimate of the effect of exposure to long working hours (41–48, 49–54 and ≥55 h/week), compared with exposure to standard working hours (35–40 h/week), on alcohol consumption (in g/week), risky drinking, and alcohol use disorder (prevalence, incidence or mortality).

**Study appraisal and synthesis methods:**

At least two review authors independently screened titles and abstracts against the eligibility criteria at a first stage and full texts of potentially eligible records at a second stage, followed by extraction of data from publications related to qualifying studies. Two or more review authors assessed the risk of bias, quality of evidence and strength of evidence, using Navigation Guide and GRADE tools and approaches adapted to this project.

**Results:**

Fourteen cohort studies met the inclusion criteria, comprising a total of 104,599 participants (52,107 females) in six countries of three WHO regions (Americas, South-East Asia, and Europe). The exposure and outcome were assessed with self-reported measures in most studies. Across included studies, risk of bias was generally probably high, with risk judged high or probably high for detection bias and missing data for alcohol consumption and risky drinking.

Compared to working 35–40 h/week, exposure to working 41–48 h/week increased alcohol consumption by 10.4 g/week (95% confidence interval (CI) 5.59–15.20; seven studies; 25,904 participants, I^2^ 71%, low quality evidence). Exposure to working 49–54 h/week increased alcohol consumption by 17.69 g/week (95% confidence interval (CI) 9.16–26.22; seven studies, 19,158 participants, I^2^ 82%, low quality evidence). Exposure to working ≥55 h/week increased alcohol consumption by 16.29 g/week (95% confidence interval (CI) 7.93–24.65; seven studies; 19,692 participants; I^2^ 82%, low quality evidence).

We are uncertain about the effect of exposure to working 41–48 h/week, compared with working 35–40 h/week on developing risky drinking (relative risk 1.08; 95% CI 0.86–1.36; 12 studies; I^2^ 52%, low certainty evidence). Working 49–54 h/week did not increase the risk of developing risky drinking (relative risk 1.12; 95% CI 0.90–1.39; 12 studies; 3832 participants; I^2^ 24%, moderate certainty evidence), nor working ≥55 h/week (relative risk 1.11; 95% CI 0.95–1.30; 12 studies; 4525 participants; I^2^ 0%, moderate certainty evidence).

Subgroup analyses indicated that age may influence the association between long working hours and both alcohol consumption and risky drinking.

We did not identify studies for which we had access to results on alcohol use disorder.

**Conclusions:**

Overall, for alcohol consumption in g/week and for risky drinking, we judged this body of evidence to be of low certainty. Exposure to long working hours may have increased alcohol consumption, but we are uncertain about the effect on risky drinking. We found no eligible studies on the effect on alcohol use disorder. Producing estimates for the burden of alcohol use disorder attributable to exposure to long working hours appears to not be evidence-based at this time.

**Protocol identifier:**

https://doi.org/10.1016/j.envint.2018.07.025.

**PROSPERO registration number:**

CRD42018084077

## Highlights

• We analysed estimates of effect of long working hours on alcohol use and disorders.
• Overall body of evidence was of low certainty.
• Exposure to long working hours potentially increased alcohol consumption.
• The effect of long working hours on risky drinking is unclear.
• We found no eligible studies on the effect on alcohol use disorder.

## 1 Background

The World Health Organization (WHO) and the International Labour Organization (ILO) are finalizing Joint Estimates of the work-related burden of disease and injury (WHO/ILO Joint Estimates) (Ryder, 2017). The organizations are estimating the numbers of deaths and disability-adjusted life years (DALYs) that are attributable to selected occupational risk factors. The WHO/ILO Joint Estimates are based on already existing WHO and ILO methodologies for estimating the burden of disease for selected occupational risk factors (Pruss-Ustun et al., 2017, International Labour Organization, 2014, ILO, 1999, Ezzati et al., 2004). They expand these existing methodologies with estimation of the burden of several prioritized additional pairs of occupational risk factors and health outcomes. For this purpose, population attributable fractions (Murray et al., 2004) are being calculated for each additional risk factor-outcome pair, and these fractions are being applied to the total disease burden envelopes for the health outcome from the WHO *Global Health Estimates* for the years 2000–2016 (World Health Organization, 2019). Population attributable fractions are the proportional reduction in burden from the health outcome achieved by a reduction of exposure to the risk factor to zero.

The WHO/ILO Joint Estimates may include estimates of the burden of alcohol consumption, risky alcohol use and alcohol use disorder attributable to exposure to long working hours if feasible, as one additional prioritized risk factor-outcome pair for which global burden of disease has not previously been estimated. To select parameters with the best and least biased evidence for our estimation models, a systematic review and meta-analysis is required of studies with estimates of the effect of exposure to long working hours on alcohol consumption, risky alcohol use and alcohol use disorder (Godderis et al., 2018). We present our findings in the current paper. WHO and ILO are in parallel also producing a systematic review of studies estimating the prevalence of exposure to long working hours (forthcoming), applying their novel systematic review methods (Pega et al., 2019). The organizations are also conducting or have completed several other systematic reviews and meta-analyses on other additional risk factor-outcome pairs (Hulshof et al., 2019, Mandrioli et al., 2018, Paulo et al., 2019, Teixeira et al., 2019, Tenkate et al., 2019, Li et al., 2018, Li et al., in press, Rugulies et al., 2019, Descatha et al., 2018, Descatha et al., in press). To our knowledge, these are the first systematic reviews and meta-analyses, with a pre-published protocol, conducted specifically for an occupational burden of disease study. The WHO’s and ILO’s joint estimation methodology and the WHO/ILO Joint Estimates are separate from these systematic reviews, and they will be described in more detail and reported elsewhere.

### 1.1 Rationale

To consider the feasibility of estimating the burden of alcohol consumption from long working hours in adherence with the guidelines for accurate and transparent health estimates reporting (GATHER) (Stevens et al., 2016), WHO and ILO require an overview of existing evidence. To achieve this, a systematic review and meta-analysis. studies was conducted with estimates of the relative effect of long working hours on alcohol consumption, risky drinking and alcohol use disorder, compared with the theoretical minimum risk exposure level. The theoretical minimum risk exposure level is defined as the exposure level that would result in the lowest possible population risk, even if it is not feasible to attain this exposure level in practice (Murray et al., 2004). These data and effect estimates derived from the current review should be tailored to serve in the future as parameters for estimating the burden of alcohol consumption, risky alcohol use and alcohol use disorder from long working hours in the WHO/ILO Joint Estimates.

To the best of our knowledge, one systematic review and meta-analysis with a similar objective was previously performed including individual participant data and cross-sectional and prospective studies (Virtanen et al., 2015). It has shown that people working long hours are more likely to use alcohol at harmful levels (odds ratio (OR) 1.11, 95% confidence interval (CI) 1.05–1.18). However, this systematic review and meta-analysis included study designs that are not acceptable for burden of disease estimation (e.g., cross-sectional studies). To our knowledge, this prior systematic review did not have a pre-published protocol and/or missed other essential aspects of a systematic review. Our systematic review is fully compliant with latest systematic review methods (including use of a protocol); includes additional outcomes crucial for burden of disease estimation (i.e., alcohol consumption, risky drinking and alcohol use disorder); and expands beyond the scope of the existing systematic review by covering evidence from studies published up to 18 April 2020.

In this systematic review, we aimed to cover workers in the formal and in the informal economy. The informal economy is defined as “all economic activities by workers and economic units that are – in law or in practice – not covered or insufficiently covered by formal arrangements”, but excluding “illicit activities, in particular the provision of services or the production, sale, possession or use of goods forbidden by law, including the illicit production and trafficking of drugs, the illicit manufacturing of and trafficking in firearms, trafficking in persons, and money laundering, as defined in the relevant international treaties” (p. 4) (104th International Labour Conference 2015). Consequently, formality of work (informal vs. formal) may be a modifier of the effect of long working hours on alcohol consumption, risky alcohol use and alcohol use disorder. Therefore, we considered studies including both formal and informal economy for inclusion in this systematic review.

### 1.2 Description of the risk factor

Burden of disease estimation requires unambiguous definition of the risk factor, risk factor levels and the theoretical minimum risk exposure level. Therefore, it is essential to define long working hours, which are the main risk factor in the current review and meta-analysis. Namely, long working hours are defined as working hours exceeding standard working hours, i.e. any working hours of ≥41 h/week (Table 1). Based on results from earlier studies on long working hours and health endpoints (e.g., Virtanen et al., 2015, Kivimäki et al., 2015), the preferred four exposure level categories for our systematic review are 35–40, 41–48, 49–54 and ≥55  h/week (Table 1).

**Table 1:** Definitions of the risk factor, risk factor levels and the minimum risk exposure level.

	Definition
Risk factor	Long working hours (including those spent in secondary jobs), defined as working hours >40 h/week, i.e. working hours exceeding standard working hours (35–40 h/week).
Risk factor levels	Four levels: 35–40 h/week. 41–48 h/week. 49–54 h/week. ≥55 h/week.
Theoretical minimum risk exposure level	Standard working hours defined as working hours of 35–40 h/week

In addition, in the context of the current review, the theoretical minimum risk exposure refers to standard working hours defined as 35–40  h/week (Table 1). We acknowledge that it is possible that the theoretical minimum risk exposure might be lower than standard working hours, but working hours <35 h/week had to be excluded because studies indicate that some individuals working less than standard hours do so because of existing health problems (Kivimäki et al., 2015, Virtanen et al., 2012). In other words, persons working less than standard hours might belong to a health-selected group or a group concerned with family care and therefore cannot serve as comparators. Consequently, if a study used subjects working less than standard hours as the reference group or a combination of subjects working standard hours and those working less than standard hours, it would be excluded from the systematic review and meta-analysis. The category 35–40  h/week was used as the reference group used in many large studies and previous systematic reviews (Virtanen et al., 2012, Kang et al., 2012).

### 1.3 Definition of the outcome

The WHO Global Health Estimates group outcomes into standard burden of disease categories (World Health Organization, 2017), based on standard codes from the *International Statistical Classification of Diseases and Related Health Problems 10th Revision* (ICD-10) (World Health Organization 2015). The first outcome of this systematic review is alcohol consumption, defined as absolute measures of total alcohol consumption in grams (g) of alcohol consumed per week (g/week), as an intermediate outcome for alcohol use disorder or potentially other disease burden categories. Whenever number of “drinks” was reported, we calculated the total amount of alcohol consumed in grams, assuming that one “drink” corresponded to 12 g of pure alcohol. We therefore applied the European Standards (10–12 g of alcohol per standard drink), but we acknowledge that this choice was somehow arbitrary and that it may have underestimated alcohol consumption for countries in which a standard drink contains more than 12 g of alcohol. The second outcome is risky drinking. We herein define risky drinking as consuming >14 drinks/week for women and >21 drinks/week for men, aligned with previous studies (Royal College of Physicians RCoGP, 1995). The other outcomes in this systematic review are prevalence of, incidence of, and mortality from alcohol use disorder. The relevant WHO Global Health Estimates category is *II.E.4 Alcohol use disorder* (ICD-10 codes: F10, G72.1, Q86.0, X45) (World Health Organization 2017). Table 2 presents each disease or health problem included in the WHO Global Health Estimates category and whether it was included in this review. This systematic review covers the entire disease burden of the relevant WHO Global Health Estimates category. Studies focusing on other alcohol-related disorders not covered in the burden of disease envelope related to alcohol-induced disorders were not included in this systematic review, to align with the WHO Global Health Estimates.

**Table 2:** ICD-10 codes and disease and health problems covered by the WHO Global Health Estimates category II.E.4 and their inclusion in the systematic review.

Alcohol use disorder		
ICD-10 Code	Disease or health problem	Included in this review
F10	Mental and behavioural disorders due to use of alcohol	Yes
G72.1	Alcoholic myopathy	Yes
Q86.0	Fetal alcohol syndrome (dysmorphic)	Yes
X45	Accidental poisoning by and exposure to alcohol	Yes

### 1.4 How the risk factor may impact the outcome

Official health estimates of the burden of disease attributable to an occupational risk factor require a sufficient level of scientific consensus that the risk factor causes the disease or other outcome (Stevens et al., 2016). A possible explanation for the association between exposure to long working hours and alcohol consumption, risky alcohol use and alcohol use disorder, respectively, is that exposure to long working hours may cause stress, and alcohol consumption may be a coping mechanism for this stress, as proposed by the tension-reduction hypothesis (Kalodner et al., 1989). However, we acknowledge that stress is a multidimensional and dynamic concept.

**Fig. 1:**
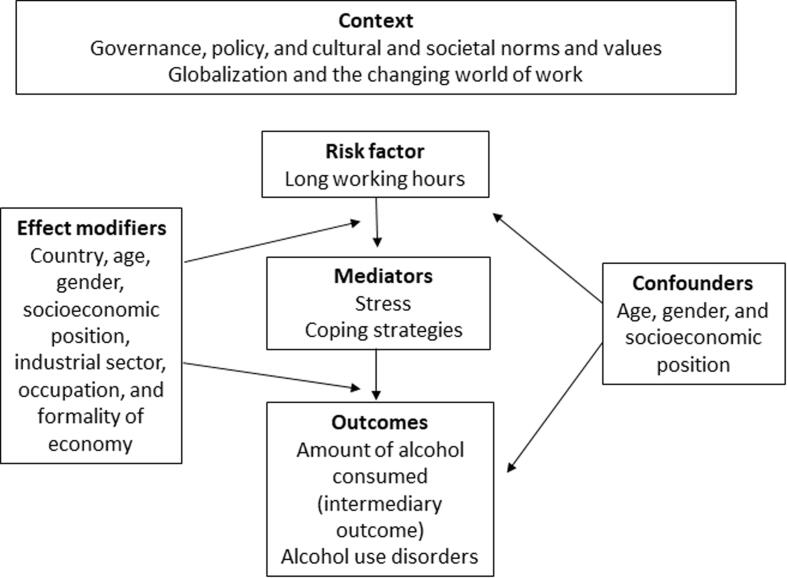
Logic model of the possible causal relationship between exposure to long working hours and alcohol consumption, risky alcohol use and alcohol use disorder.

Source: Godderis et al., 2018.

Fig. 1 presents the logic model for the causal relationship between exposure to long working hours and alcohol consumption, risky alcohol use and alcohol use disorder, respectively. This is an *a priori*, process-oriented model (Rehfuess et al., 2017) that seeks to capture the complexity of the causal relationship between exposure to long working hours and alcohol consumption and alcohol use disorder, respectively. We assume that the effect of exposure to long working hours on alcohol consumption, risky alcohol use and alcohol use disorder could be modified by country, age, sex, socioeconomic position, industrial sector, occupation and/or formality of economy. Confounding should be considered by age, sex and socioeconomic position (e.g. income, education or occupational grade). We also assume that the effects of long working hours on alcohol consumption, risky alcohol use and alcohol use disorder are mediated through two pathways, namely work-related stress imposed by long working hours and individual coping strategies, herein defined as the individual worker’s ability to deal with stress and anxiety derived from job demands and especially long working hours (Barnes, 2014, Bartone et al., 2017, Corbin et al., 2013, Park et al., 2014).

Rodent models also support a causal effect of external stress on alcohol consumption. Interactions between stress and the reward system seem to induce alcohol consumption, especially in alcohol-experienced people. Glucocorticoids effects within the nucleus accubems, which plays an important role in the cognitive processing of motivation and reward, are likely mediators in this relationship. An increased activation of the corticotrophin-releasing hormone (CRH) within the amigdala has been also implicated. After they have been exposed to different types of stressors, rats increase alcohol consumption with a delay that parallels the one observed in humans relapsing to heavy alcohol use after a stressful period. This body of evidence is related to stress rather than to long working hours *per se*, and, therefore, it should be regarded only as indirect evidence of a causal relationship between exposure with long working hours and alcohol consumption, and perhaps also with risky alcohol use and alcohol use disorder (Liu and Weiss, 2003, Noori et al., 2014, Spanagel et al., 2014).

## 2 Objectives

To systematically review and meta-analyse evidence on the effect of exposure to long working hours (three categories: 41–48, 49–54 and ≥55 h/week) on alcohol consumption, risky alcohol use and alcohol use disorder prevalence, incidence and mortality among workers of working age, compared with the minimum risk exposure level (standard working hours: 35–40 h/week).

## 3 Methods

### 3.1 Developed protocol

We applied the Navigation Guide systematic review methodology for systematic reviews in occupational and environmental health as our guiding methodological framework, wherever feasible (Woodruff and Sutton, 2014). The guide applies established systematic review methods from clinical medicine, including standard Cochrane methods for systematic reviews of interventions, to the field of occupational and environmental health to ensure systematic and rigorous evidence synthesis on occupational and environmental risk factors that reduces bias and maximizes transparency (Woodruff and Sutton, 2014). The need for further methodological development and refinement of the relatively novel Navigation Guide has been acknowledged (Woodruff and Sutton, 2014). From the perspective of the Navigation Guide framework, all steps were conducted (i.e., steps 1–6 in Fig. 1 in (Woodruff and Sutton, 2014) for the stream on human data and none of the steps for the stream on non-human data, although we narratively synthesized the mechanistic evidence from non-human data that we were aware of (Section 1.4.).

We have prospectively registered the protocol in the International Prospective Register of Systematic Reviews, PROSPERO, under CRD42018084077. The protocol adheres to the Preferred Reporting Items for Systematic Review and Meta-Analysis protocols statement (PRISMA-P) (Moher et al., 2015, Shamseer et al., 2015), with the abstract adhering to the Preferred Reporting Items for Systematic Review and Meta-Analysis in journal and conference abstracts (PRISMA-A) (Beller et al., 2013). Any modification of the methods stated in the protocol was registered in PROSPERO and reported in the systematic review itself (Section 8). Our systematic review is reported according to the preferred reporting items for systematic review and meta-analysis statement (PRISMA) (Liberati et al., 2009). We planned to report the parameters for estimating the burden of alcohol consumption to long working hours in the systematic review in accordance with the requirements of the GATHER guidelines (Stevens et al., 2016). This would be done because the WHO/ILO burden of disease estimates that could be produced following the systematic review must also adhere to these reporting guidelines.

### 3.2 Inclusion criteria

The PECO (Liberati et al., 2009) criteria are described below.

#### 3.2.1 Types of populations

We included studies of the working-age population (≥15 years) in the formal and informal economy. Studies of children (aged < 15 years) and unpaid domestic workers were excluded. Participants residing in any WHO and/or ILO Member State and any industrial setting or occupational group were included. Exposure to long working hours may potentially have further population reach (e.g., across generations for workers of reproductive age) and acknowledged that the scope of our systematic review does not capture these populations and impacts on them. Our protocol paper provides a complete, but briefer overview of the PECO criteria (Godderis et al., 2018).

#### 3.2.2 Types of exposures

We included studies that defined long working hours in accordance with our standard definition (Table 1). We again prioritized measures of the total number of hours worked, including in both of: main and secondary jobs, self-employment and salaried employment, whether in the informal or the formal economy. We included studies with objective (e.g., by means of time recording technology) or subjective measurements of long working hours, whether, including studies that used measurements by experts (e.g., scientists with subject matter expertise) and self-reports by the worker, workplace administrator or manager. We planned to prioritize objective measurements for studies that have presented both objective and subjective measurements. Studies with measures from any data source, including registry data, were included. For studies that reported exposure levels differing from our standard levels (Table 1), we converted the reported levels to the standard levels and reported analyses on these alternate exposure levels if impossible.

#### 3.2.3 Types of comparators

The included comparator were participants exposed to the theoretical minimum risk exposure level: worked 35–40 h/week (Table 1).

#### 3.2.4 Types of outcomes

We included studies that defined alcohol consumption, risky alcohol use and alcohol use disorders in accordance with our standard definition of these outcomes or for which re-analyses of individual participant data were feasible. We included studies that assessed absolute measures of alcohol consumption in grams of alcohol consumed per average week (outcome 1), risky drinking (outcome 2), and/or the prevalence of, incidence of or mortality from any alcohol use disorder, as defined by the ICD-10 codes F10, G72.1, Q86.0, and/or X45 (outcome 3) (Table 2). For alcohol consumption, we considered for inclusion studies that measured the outcome using validated tools (e.g. AUDIT-C) (Bradley et al., 1998) or other self-reporting by means of questionnaire. For alcohol use disorder, we considered for inclusion studies with documented International Classification of Diseases-10 (ICD-10) diagnostic codes and studies that applied methods to ascertain the diagnosis of alcohol use disorder per ICD-10 criteria.

The following measurements of alcohol use disorder were regarded as eligible:

(i) Diagnosis by a physician.
(ii) Hospital discharge record.
(iii) Other relevant administrative records (e.g. records of sickness absence or disability).
(iv) Medically certified cause of death.

All other measures were not considered for inclusion in this systematic review.

We planned to include both objective and subjective measures of this outcome but to prioritize objective over subjective ones.

#### 3.2.5 Types of studies

We included studies that investigated the effect of long working hours on alcohol consumption, risky alcohol use or alcohol use disorder for any years. Eligible study designs were randomized controlled trials (including parallel-group, cluster, cross-over and factorial trials), cohort studies (both prospective and retrospective), case-control studies, and other non-randomized intervention studies (including quasi-randomized controlled trials, controlled before-after studies and interrupted time series studies). We included a broader set of observational study designs than is commonly included, because a recent augmented Cochrane Review of complex interventions identified valuable additional studies using such a broader set of study designs (Arditi et al., 2016). As we have an interest in quantifying risk and not in qualitative assessment of hazard (Barroga and Kojima, 2013), all other study designs were excluded (e.g., uncontrolled before-and-after, cross-sectional, qualitative, modelling, case and non-original studies).

Records published in any year and any language were included. The search was conducted using English language terms, so that records published in any language that present essential information (i.e. title and abstract) in English could be included. We planned to translate records written in a language other than those spoken by the authors of this review or those of other reviews (Descatha et al., in press; Hulshof et al., Under review; John et al., Under review; Li et al., in press, Mandrioli et al., 2018; Rugulies et al., Under review; Teixeira et al., Under review; Tenkate et al., Under review) in the series (i.e. Arabic, Bulgarian, Chinese, Danish, Dutch, English, French, Finnish, German, Hungarian, Italian, Japanese, Norwegian, Portuguese, Russian, Spanish, Swedish and Thai), then we translated the record into English. Published and unpublished studies were considered for inclusion. Studies conducted using unethical practices were excluded (e.g., studies that deliberately exposed humans to a known risk factor to human health).

#### 3.2.6 Types of effect measures

We considered for inclusion measures of the relative effect of a relevant level of long working hours on alcohol consumption, risky alcohol use and/or the risk of alcohol use disorder (prevalence, incidence and mortality), compared with the theoretical minimum risk exposure level. Relative effect measures considered for inclusion were mean differences, for alcohol consumption, risk ratios and odds ratios for risky drinking and prevalence and mortality measures, and hazard ratios for incidence measures (e.g. developed or died from an alcohol use disorder). To ensure comparability of effect estimates and facilitate meta-analysis, if a study presented an OR, we planned to convert it into a risk ratio, if possible, using the guidance provided in Cochrane’s Handbook for Systematic Reviews of Interventions (Cumpston et al., 2019).

As shown in our logic model (Fig. 1), we *a priori* considered the following variables to be potential effect modifiers of the effect of long working hours on alcohol consumption, risky alcohol use and on alcohol use disorder: country, age, sex, industrial sector, occupation, and formality of employment. We considered age, sex, and socioeconomic position to be potential confounders. Potential mediators were work-related stress imposed by long-working hours and the individual worker’s specific coping strategies.

If a study presented estimates for the effect from two or more alternative models that have been adjusted for different variables, we planned to prioritize the estimate from the model that we consider best adjusted, applying the lists of confounders and mediators identified in our logic model (Fig. 1). We planned to prioritize estimates from models adjusted for more potential confounders over those from models adjusted for fewer. We would prioritize estimates from models unadjusted for mediators over those from models that adjusted for mediators, because adjustment for mediators can introduce bias. If a study presented effect estimates from two or more potentially eligible models, we planned to explain specifically why we prioritized the selected model.

### 3.3 Searched literature

#### 3.3.1 Electronic bibliographic databases

We searched the seven following electronic bibliographic databases:

1. International Clinical Trials Register Platform (to 30 June 2018).
2. Ovid MEDLINE with Daily Update (1 January 1946–30 June 2018).
3. PubMed (1 January 1946–18 April 2020).
4. EMBASE (1 January 1947–30 June 2018).
5. Web of Science (1 January 1945–30 June 2018).
6. CISDOC (1 January 1901–2012).
7. PsychInfo (1 January 1880–30 June 2018).

The Ovid MEDLINE search strategy was presented in the protocol (Godderis et al., 2018a, Godderis et al., 2018b). The full search strategies for all databases were revised by an information scientist and are presented in Appendix 2 in the Supplementary data. When we neared completion of the review, we conducted a top-up search of the MEDLINE database on April 2020 to capture the most recent publications (e.g., publications ahead of print).

#### 3.3.2 Electronic grey literature databases

We searched the two following electronic databases for grey literature:

1. OpenGrey (http://www.opengrey.eu/)
2. Grey Literature Report (http://greylit.org/)

#### 3.3.3 Internet search engines

We also searched the Google (www.google.com/) and Google Scholar (www.google.com/scholar/) Internet search engines and screened the first 100 hits for potentially relevant records, as has been done previously in Cochrane Reviews (Pega et al., 2015, Pega et al., 2017).

#### 3.3.4 Organizational websites

The websites of the seven following international organizations and national government departments were searched on June 2018:

1. International Labour Organization (www.ilo.org/).
2. World Health Organization (www.who.int).
3. European Agency for Safety and Health at Work (https://osha.europa.eu/en).
4. Eurostat (www.ec.europa.eu/eurostat/web/main/home).
5. China National Knowledge Infrastructure (http://www.cnki.net/).
6. Finnish Institute of Occupational Health (https://www.ttl.fi/en/).
7. United States National Institute of Occupational Safety and Health (NIOSH) of the United States of America, using the NIOSH data and statistics gateway (https://www.cdc.gov/niosh/data/).

#### 3.3.5 Hand-searching and expert consultation

We hand-searched for potentially eligible studies in:

• Reference list of previous systematic reviews.
• Reference list of all included study records.
• Study records published over the past 24 months in the three peer-reviewed academic journals with the largest number of included studies.
• Study records that have cited the included studies (identified in Web of Science citation database).
• Collections of the review authors.

### 3.4 Selected studies

Study selection was carried out with the Covidence software (Babineau, 2014). All study records identified in the search were downloaded and duplicates were identified and deleted. Afterwards, at least two review authors, working in pairs, independently screened titles and abstracts (step 1) and then full texts (step 2) of potentially relevant records. A third review author resolved any disagreements between the two review authors. We planned to assign study records authored by a review author to a review author not involved in the study. The study selection was documented in a flow chart in the systematic review, as per PRISMA guidelines (Liberati et al., 2009).

### 3.5 Extracted data

A standard data extraction form was developed and trialled until data extractors reached convergence and agreement. At least two review authors independently extracted data on study characteristics (including study authors, study year, study country, participants, exposure, and outcome), study design (including study type, comparator, epidemiological model(s) used, and effect estimate measure) and risk of bias (including source population representation, blinding, exposure assessment, outcome assessment, confounding, incomplete outcome data, selective outcome reporting, conflict of interest, and other sources of bias). A third review author resolved conflicts in data extraction. Data were entered into and managed with Microsoft Excel.

We also extracted data on potential conflict of interest in included studies. For each author and affiliated organization of each included study record, we extracted their financial disclosures and funding sources. We used a modification of a previous method to identify and assess undisclosed financial interest of authors (Forsyth et al., 2014). Where no financial disclosure or conflict of interest statements were available, we searched the name of all authors in other study records gathered for this study and published in the prior 36 months and in other publicly available declarations of interests (Drazen et al., 2010a, Drazen et al., 2010b)

### 3.6 Requested missing data

We requested missing data from the principal study author by email or phone, using the contact details provided in the principal study record. If we did not receive a positive response from the study author, follow-up emails were sent twice, at two and four weeks. We present a description of missing data, the study author from whom the data were requested, the date of requests sent, the date on which data were received (if any), and a summary of the responses provided by the study authors (**Appendix 3** in the Supplementary data). If we did not receive some or all requested missing data, we nevertheless retained the study in the systematic review as long as it fulfilled our eligibility criteria.

### 3.7 Methodology used to analyse individual participant data (IPD) from unpublished studies

We obtained data of relevant datasets from IPD collections with measurements of working hours and alcohol consumption. We considered working hours/week as hours worked in all current jobs including hours worked at home, if specified in the data set. Participants who worked <35 h/week at baseline or during follow up were excluded, as well as participants younger than 15 years old. The categorization of hours/week was as follows: 35–40 (reference), 41–48, 49–54 and ≥55 h. Alcohol consumption was defined as absolute measures of alcohol in grams per week. Alcohol consumption was calculated from the questions on frequency of alcohol use and on the amount of drinks over a time period. A standard drink was considered to contain 12 g of pure alcohol. Sex, age and socio-economic status (SES) were included as confounding factors. Age categories for stratification were 15–29 years, 30–44 years, 45–59 years and ≥60 years at baseline. SES was inferred from income, whenever possible, or alternatively from education level (primary, secondary and higher). Income categories were the three income tertiles of the population if income was registered as a continuous variable or as per the income categories used at baseline. Mean alcohol consumption per week with standard deviation was calculated for the total population and stratified by gender, age and SES at different follow up time points.

Multiple log-binomial regression models were used to assess the relationship between working hours and risky drinking, while adjusting for sex, age and SES. All analyses were disaggregated by sex (female or male) and different 5-year age groups (15–19, 20–24, 25–29, 30–34, 35–39, 40–44, 45–49, 50–54, 55–59, 60–64, and over 65 years old). Sex disaggregated analyses were only adjusted for age and SES, while age disaggregated analyses were only adjusted for sex and SES. Working hours were dummy-coded with 35–40 h/w as the reference group. Income was median-centered per year to control for possible inflation-effects. Age was mean-centered to avoid convergence problems and sex was dummy-coded with males as the reference group. All statistical analyses were conducted in R (version 3.3.1) with package logbin (version 2.0.4).

### 3.8 Assessed risk of bias

There are no standardized risk of bias tools for systematic reviews for hazard identification or those for risk assessment in occupational and environmental health. Nonetheless, there are five existing methods in this field specifically developed for hazard identification or/and risk assessment, and they differ substantially in the types of studies (randomized, observational and/or simulation studies) and data (e.g. human, animal and/or *in vitro*) they seek to assess (Rooney et al., 2016). However, all five methods, including the *Navigation Guide, which we used as our organizing framework*, use a similar approach to assess risk of bias in human studies (Rooney et al., 2016).

Therefore, to remain consistent, we used the Navigation Guide risk of bias tool, which builds on the standard risk of bias assessment methods of Cochrane (Higgins et al., 2011) and the US Agency for Healthcare Research and Quality (Viswanathan et al., 2008). Some further refinements of the Navigation Guide method may be warranted (Goodman et al., 2017), but it has been successfully applied in several completed and ongoing systematic reviews (Johnson et al., 2014, Johnson et al., 2016, Koustas et al., 2014, Lam et al., 2014, Lam et al., 2016, Lam et al., 2017, Lam et al., 2016, Vesterinen et al., 2014). In our application of the Navigation Guide method, we draw heavily on one of its latest versions, as presented in the protocol for an ongoing systematic review (Lam et al., 2016).

Risk of bias was assessed on the individual study level and across the entire body of evidence for each outcome. The nine risk of bias domains assessed were: (i) source population representation; (ii) blinding; (iii) exposure assessment; (iv) outcome assessment; (v) confounding; (vi) incomplete outcome data; (vii) selective outcome reporting; (viii) conflict of interest; and (ix) other sources of bias. Risk of bias or confounding ratings for all domains were: “low”; “probably low”; “probably high”; “high” or “not applicable” (Lam et al., 2016). To judge the risk of bias in each domain, we applied a priori instructions (Godderis et al., 2018), which were adapted from an ongoing Navigation Guide systematic review (Lam et al., 2016), and further described in our protocol (Godderis et al., 2018). For example, a study was assessed as carrying “low” risk of bias from source population representation, if we judge the source population to be described in sufficient detail (including eligibility criteria, recruitment, enrolment, participation and loss to follow up) and the distribution and characteristics of the study sample to indicate minimal or no risk of selection effects.

All risk of bias assessors jointly trialled the application of the risk of bias criteria until they had synchronized their understanding and application of these criteria. Two or more study authors independently assessed the risk of bias for each study by outcome. In case of discrepancies, a third author resolved the conflict. For each included study, we reported our risk of bias assessment at the level of the individual study by domain in a standard ‘Risk of bias table’ (Higgins et al., 2011). For the entire body of evidence, we presented the study-level risk of bias ratings by domains in a ‘Risk of bias summary figure’ (or ‘Risk of bias matrix’) (Higgins et al., 2011). As we only included unpublished studies, we searched for information necessary to assess risk of bias in related study records (if any), the study website and/or the study codebook.

### 3.9 Synthesised evidence (including conducted meta-analysis)

We planned to conduct meta-analyses separately for estimates of the effect of the exposure on alcohol consumption, risky alcohol use and alcohol use disorders (prevalence, incidence and mortality). We planned to not combine studies of different designs (e.g. combining cohort studies with case-controls studies) quantitatively. Cases of inconsistency of effect estimates across studies were investigated to identify sources of clinical heterogeneity in terms of participants (including country, sex, age and industrial sector or occupation). Effect estimates differing considerably by country, sex and/or age, or a combination of these were synthesised for the relevant populations defined by country, sex and/or age, or combination thereof. Differences by country were expanded to include differences by WHO region. Effect estimates clinically homogenous across countries, sexes and age groups were combined into one pooled effect estimate that could be applied across all combinations of countries, sexes and age groups in the WHO/ILO joint methodology.

When two or more studies for the relevant combination of country, sex and age group, or combination thereof, were considered sufficiently clinically homogenous to potentially be combined quantitatively using quantitative meta-analysis we tested the statistical heterogeneity of the studies using the I^2^ statistic. If two or more clinically homogenous studies were found to be sufficiently homogenous statistically to be combined in a meta-analysis, we will pool the risk ratios of the studies in a quantitative meta-analysis, using the inverse variance method with a random effects model to account for cross-study heterogeneity. Meta-analyses were conducted in RevMan 5.3, but the data for entry into these programmes was prepared using R. We planned to not quantitatively combine data from unadjusted and adjusted models.

### 3.10 Additional analyses

Subgroup analyses were conducted only for the main meta-analysis and comparison of interest (i.e., the meta-analysis of cohort studies for the comparison of worked ≥55 h/week versus worked 35–40 h/week). We conducted subgroup analyses by:

• WHO region.
• Sex.
• Age group.
• SES.

We also planned to conduct subgroup analyses by occupation, industrial sector and formality of economy, but did not find evidence or receive missing data to populate these subgroup analyses.

We planned to conduct the following sensitivity analyses:

• Studies judged to be of “high”/“probably high” risk of bias in any domain, compared with “low”/“probably low” risk of bias in all domains.
• Studies with documented or approximated ICD-10 diagnostic codes (e.g., as recorded in administrative health records), compared with studies without ICD-10 diagnostic codes (e.g., self-reports).
• Studies with “low” or “probably low” risk of bias from conflict of interest with studies with any “high” or “probably high” risk of bias in this domain.

However, we did not conduct any of these sensitivity analyses, because all included studies fell in the same category in each sensitivity analysis.

### 3.11 Assessed quality of evidence

We assessed quality of evidence using a modified version of the Navigation Guide quality of evidence assessment tool (Lam et al., 2016). The tool is based on the Grading of Recommendations Assessment, Development and Evaluation (GRADE) approach (Schünemann et al., 2011) adapted specifically to systematic reviews in occupational and environmental health (Morgan et al., 2016).

At least two review authors assessed quality of evidence for the entire body of evidence by outcome, with any disagreements resolved by a third review author. We adapted the latest *Navigation Guide* instructions (Godderis et al., 2018) for grading the quality of evidence. We downgraded the quality of evidence for the following five GRADE reasons: (i) risk of bias; (ii) inconsistency; (iii) indirectness; (iv) imprecision; and (v) publication bias. We generated a funnel plot to judge publication bias for meta-analyses that included ten or more studies and judged the risk of publication bias qualitatively for meta-analyses with nine or fewer studies.

We graded the evidence, using the three *Navigation Guide* standard quality of evidence ratings: “high”, “moderate” and “low”. Within each of the relevant domains, we rated the concern for the quality of evidence, using the ratings “none”, “serious” and “very serious”. As per *Navigation Guide*, we started at “high” for randomized studies and “moderate” for observational studies. Quality was downgrade for no concern by nil grades (0), for a serious concern by one grade (-1) and for a very serious concern by two grades (-2). We considered up-grading the quality of evidence for the following reasons: large effect, dose–response and plausible residual confounding and bias. For example, if we had a serious concern for risk of bias in a body of evidence consisting of observational studies (-1), but no other concerns, and there are no reasons for upgrading, then we would downgrade its quality of evidence by one grade from “moderate” to “low”.

### 3.12 Assessed strength of evidence

Our systematic review included observational epidemiologic studies of human data only, and no other streams of evidence (e.g., no studies of non-human data). The standard Navigation Guide methodology (Lam et al., 2016) allows for rating human and non-human animal studies separately, and then combining the strength of evidence for each stream for an overall strength of evidence rating. However, the Navigation Guide also allows for rating one stream of evidence based on the factors described above (i.e., risk of bias, indirectness, inconsistency, imprecisions, publication bias, large magnitude of effect, dose–response and residual confounding) to arrive at an overall rating of the quality of evidence as ‘high’, ‘moderate’ or ‘low’ (see above and the protocol). The approach of evaluating only the human evidence stream is consistent with the GRADE methodology that has adopted the Bradford Hill considerations (Schunemann et al., 2011). So, using the method above based on the Navigation Guide incorporates the considerations of Bradford Hill (Table 3).

**Table 3:** Bradford Hill considerations and their relationship to GRADE and the Navigation Guide for evaluating the overall quality of the evidence for human observational studies.

Bradford Hill	GRADE	Navigation Guide
Strength	Strength of association and imprecision in effect estimate	Strength of association and imprecision in effect estimate
Consistency	Consistency across studies, i.e., across different situations (different researchers)	Consistency across studies, i.e., across different situations (different researchers)
Temporality	Study design, properly designed and conducted observational studies	Study design, properly designed and conducted observational studies
Biological Gradient	Dose response gradient	Dose response gradient
Specificity	Indirectness	Indirectness
Coherence	Indirectness	Indirectness
Experiment	Study design, properly designed and conducted observational studies	Study design, properly designed and conducted observational studies
Analogy	Existing association for critical outcomes leads to not downgrading the quality, indirectness	Existing association for critical outcomes leads to not downgrading the quality, indirectness. Evaluating the overall strength of body of human evidence allows consideration of other compelling attributes of the data that may influence certainty.

Table adapted from (Schunemann et al., 2011).

There is an additional step that is described in the protocol that integrates the quality of the evidence (method for assessing described above) with other elements including direction of effect and confidence in the effect and other compelling attributes of the data. These attributes may influence our certainty to allow for an overall rating that consists of “sufficient evidence of toxicity/harmfulness”, “limited of toxicity/harmfulness”, “inadequate of toxicity/harmfulness” and “evidence of lack of toxicity/harmfulness” based on human evidence. This approach to evaluate only the human evidence has been applied in previous systematic reviews and verified by the US National Academy of Sciences (National Academies of Sciences, 2017). It also provides two steps that integrate Bradford Hill criteria (evaluating the quality of the evidence and then evaluating the overall strength of evidence). Finally, the GRADE quality of evidence ratings (which are the same as for Navigation Guide) are analogous to the final ratings from Bradford Hill for causality which has been described in Schunemann et al. (2011) (Table 4).

**Table 4:** Interpretation of the GRADE ratings of the overall quality of evidence and the Navigation Guide ratings for strength of evidence evaluation.

GRADE rating for quality of evidence	Interpretation of GRADE rating	Navigation Guide rating for strength of evidence for human evidence	Interpretation of Navigation Guide rating
**High**	There is high confidence that the true effect lies close to that of the estimate of the effect.	**Sufficient evidence of toxicity**	A positive relationship is observed between exposure and outcome where chance, bias, and confounding can be ruled out with reasonable confidence. The available evidence includes results from one or more well-designed, well conducted studies, and the conclusion is unlikely to be strongly affected by the results of future studies.
**Moderate**	There is moderate confidence in the effect estimate: the true effect is likely to be close to the estimate of the effect, but there is a possibility that it is substantially different.	**Limited evidence of toxicity**	A positive relationship is observed between exposure and outcome where chance, bias, and confounding cannot be ruled out with reasonable confidence. Confidence in the relationship is constrained by such factors as: the number, size, or quality of individual studies, or inconsistency of findings across individual studies. As more information becomes available, the observed effect could change, and this change may be large enough to alter the conclusion.
**Low**	The panel’s confidence in the effect estimate is limited: the true effect may be substantially different from the estimate of the effect	**Inadequate evidence of toxicity**	The available evidence is insufficient to assess effects of the exposure. Evidence is insufficient because of the limited number or size of studies, low quality of individual studies, or inconsistency of findings across individual studies. More information may allow an assessment of effects.
**Very Low**	There is little confidence in the effect estimate: the true effect is likely to be substantially different from the estimate of effect.		

Adapted from (Schunemann et al., 2011) and (Lam et al., 2016).

## 4 Results

### 4.1 Study selection

**Fig. 2:**
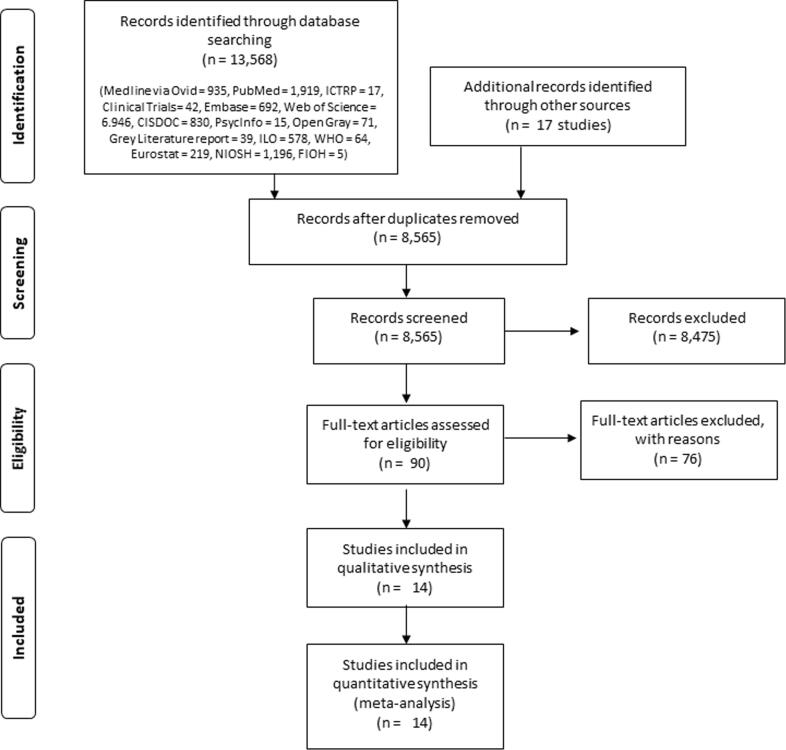
Study flow diagram.

Of the total of 8565 individual study records identified in our searches, 14 studies fulfilled the eligibility criteria and were included in the systematic review (Fig. 2). For the 76 excluded studies that most closely resembled inclusion criteria, the reasons for exclusion are listed in **Appendix 4** in the Supplementary data. All fourteen studies were included in one or more quantitative meta-analyses.

### 4.2 Characteristics of included studies

The characteristics of the included studies are summarized in Table 5.

**Table 5:** Characteristics of included studies.

Part I: Study population and study type										
**Study**	**Study population**							**Study type**		
Study ID	Total number of study participants	Number of female study participants	Country of study population	Geographic location	Industrial sector (ISIC-4)	Occupation (ISCO-08)	Age	Study design	Study period (month of first collection of any data and month of last collection of any data)	Follow-up period (period in months between exposure and outcome)
ACL	1502	802	United States	National	Multiple	Multiple	Mean age 44.5 years at baseline	Prospective cohort study	1986 (baseline) to 2002	16 years
Alameda	1585	666	United States	Regional	Multiple	Multiple	Mean age 44.4 years at baseline	Prospective cohort study	1973 (baseline) to 1994	20 years
BCS	17,196	8279	United Kingdom	National	Multiple	Multiple	Participants followed from birth	Prospective cohort study	1970 to present	49 years
BELSTRESS	2821	871	Belgium	National	Multiple	Multiple	Mean age 45.8	Prospective cohort study	1995–2003	8 years
HeSSup	12,380	7293	Finland	National	Multiple	Multiple	Mean age 39.6	Prospective cohort study	1998–2012	15 years*
HILDA	2269	712	Australia	National	Multiple	Multiple	Mean age 39.7	Prospective cohort study	2002–2017	15 years
MIDUS	3303	1637	United States	National	Multiple	Multiple	Mean age 44.2 years at baseline	Prospective cohort study	1995 (baseline) to 2005	10 years
NCDS	17,416	8708	United Kingdom	National	Multiple	Multiple	Participants followed from birth	Prospective cohort study	1958- to date	62 years
NHANES I	3794	1880	United States	National	Multiple	Multiple	Mean age 47.7	Prospective cohort study	1982–1992	10 years
NLSY79	4787	2208	United States	National	Multiple	Multiple	Mean age 41.4	Prospective cohort study	2002–2014	12 years
NSFH	13,017	6508	United States	National	Multiple	Multiple	Not clear	Prospective cohort study	1987–2003	16 years
SOEP	16698**	8349	Germany	National	Multiple	Multiple	Mean age 42.8	Prospective cohort study	1984–2016	30 years***
WLSG	5421	2883	United States	Local (Wisconsin)	Multiple	Multiple	Mean age 54.1 years at baseline	Prospective cohort study	1992 (baseline) to 2003–2005	10 years
WLSS	2366	1299	United States	Local (Wisconsin)	Multiple	Multiple	Mean age 52.4 years at baseline	Prospective cohort study	1993 (baseline) to 2004–2007	12 years

*Part II: exposure assessment and comparator*								
**Study**	**Exposure assessment**							**Co-exposure with other occupational risk factors**
Study ID	Exposure definition	Unit for which exposure was assessed	Mode of exposure data collection	Exposure assessment methods	Levels/intensity of exposure	Number of study participants in exposed group	Number of study participants in unexposed group	Potential co-exposure with other occupational risk factors
ACL	Weekly working hours	Individual level	Survey	Self-reported	35–40 h/week, 41–48 h/week, 49-54 h/week, ≥55 h/week	495	Unclear	Unknown, probably multiple
Alameda	Weekly working hours	Individual level	Survey	Self-reported	35–40 h/week, 41–48 h/week, 49-54 h/week, ≥55 h/week	265	Unclear	Unknown, probably multiple
BCS	Weekly working hours	Individual level	Interview	Self-reported	35–40 h/week, 41–48 h/week, 49-54 h/week, ≥55 h/week	881	Unclear	Unknown, probably multiple
Belstress	Weekly working hours	Individual level	Survey	Self-reported	35–40 h/week, 41–48 h/week, 49-54 h/week, ≥55 h/week	670	1560	Unknown, probably multiple
HeSSup	Weekly working hours	Individual level	Survey	Self-reported	35–40 h/week, 41–48 h/week, 49-54 h/week, ≥55 h/week	6065	2894	Unknown, probably multiple
HILDA	Weekly working hours	Individual level	Interview	Self-reported	35–40 h/week, 41–48 h/week, 49-54 h/week, ≥55 h/week	1309	960	Unknown, probably multiple
MIDUS	Weekly working hours	Individual level	Survey	Self-reported	35–40 h/week, 41–48 h/week, 49-54 h/week, ≥55 h/week	1239	Unclear	Unknown, probably multiple
NCDS	Weekly working hours	Individual level	Survey	Self-reported	35–40 h/week, 41–48 h/week, 49-54 h/week, ≥55 h/week	1103	Unclear	Unknown, probably multiple
NHANES I	Weekly working hours	Individual level	Survey	Self-reported	35–40 h/week, 41–48 h/week, 49-54 h/week, ≥55 h/week	1358	2436	Unknown, probably multiple
NLSY79	Weekly working hours	Individual level	Interview	Self-reported	35–40 h/week, 41–48 h/week, 49-54 h/week, ≥55 h/week	2002	2785	Unknown, probably multiple
NSFH	Weekly working hours	Individual level	Interview	Self-reported	35–40 h/week, 41–48 h/week, 49-54 h/week, ≥55 h/week	862	Unclear	Unknown, probably multiple
SOEP	Weekly working hours	Individual level	Survey	Self-reported	35–40 h/week, 41–48 h/week, 49-54 h/week, ≥55 h/week	5164	4665	Unknown, probably multiple
WLSG	Weekly working hours	Individual level	Survey	Self-reported	35–40 h/week, 41–48 h/week, 49-54 h/week, ≥55 h/week	724	4697	Unknown, probably multiple

Part III: outcome assessment and statistical modelling										
**Study**	**Outcome assessment**									**Comparator**
Study ID	Definition of outcome	Which International Classification of Diseases (ICD) code was reported for the outcome (if any)?	Method of outcome assessment	Diagnostic assessment method	Specification of outcome	Number of cases with outcome of interest in exposed group	Number of non-cases (i.e. without outcome of interest) in exposed group	Number of cases with outcome of interest in unexposed group	Number of non-cases (i.e. without outcome of interest) in unexposed group	Definition of comparator
ACL	Alcohol units per month	No	Interview	Not applicable	Risky drinking	Not applicable	Not applicable	Not applicable	Not applicable	35–40 hours/week
Alameda	Frequency and quantity of alcohol consumption per month	No	Questionnaire	Not applicable	Risky drinking	Not applicable	Not applicable	Not applicable	Not applicable	35–40 hours/week
BCS	Alcohol units per week	No	Interview	Not applicable	Risky drinking	Not applicable	Not applicable	Not applicable	Not applicable	35–40 hours/week
Belstress	Average number of alcohol units consumed on week days and weekend days	No	Standardized questionnaire	Not applicable	Alcohol consumption in g/week	Not applicable (outcome: alcohol consumption in g/week)	Not applicable (outcome: alcohol consumption in g/week)	Not applicable (outcome: alcohol consumption in g/week)	Not applicable (outcome: alcohol consumption in g/week)	35–40 hours/week
HeSSup	Alcohol consumption in g/week	No	Self-reported survey	Not applicable	Alcohol consumption in g/week	Not applicable (outcome: alcohol consumption in g/week)	Not applicable (outcome: alcohol consumption in g/week)	Not applicable (outcome: alcohol consumption in g/week)	Not applicable (outcome: alcohol consumption in g/week)	35–40 hours/week
HILDA	Frequency and amount of alcohol consumption	No	Self-reported	Not applicable	Risky drinking defined as >21 drinks/week for men and > 14 drinks/week for women	121	761	57	517	35–40 hours/week
MIDUS	Frequency and amount of alcohol consumption	No	Self-reported	Not applicable	Risky drinking	Not applicable	Not applicable	Not applicable	Not applicable	35–40 hours/week
NCDS	Units of alcohol consumed per week	No	Self-reported	Not applicable	Measures on frequency and number of alcoholic drinks converted to units of alcohol consumed per week	Not applicable	Not applicable	Not applicable	Not applicable	35–40 hours/week
NHANES	Frequency and amount of alcohol consumption	No	Self-reported	Not applicable	Risky drinking defined as >21 drinks/week for men and > 14 drinks/week for women	34	767	51	1150	35–40 hours/week
NLSY79	Frequency and amount of alcohol consumption	No	Self-reported	Not applicable	Alcohol consumption in g/week	28	1336	45	1639	35–40 hours/week
NSFH	Frequency and amount of alcohol consumption	No	Self-reported	Not applicable	Risky drinking	Not applicable	Not applicable	Not applicable	Not applicable	35–40 hours/week
SOEP	Alcohol consumption in g/week	No	Self-reported	Not applicable	Alcohol consumption in g/week	Not applicable	Not applicable	Not applicable	Not applicable	35–40 hours/week
WLSG	Number of drinks consumed last month	No	Self-reported	Not applicable	Alcohol consumption in g/week Risky drinking	Not applicable	Not applicable	Not applicable	Not applicable	35–40hours/week
WLSS	Number of drinks consumed last month	No	Self-reported	Not applicable	Alcohol consumption in g/week Risky drinking	Not applicable	Not applicable	Not applicable	Not applicable	35–40 hours/week

* Included data on alcohol consumption was assessed in the 1998 survey.

**(Samples A, B, C).

*** Alcohol consumption once in 2016.

Information on the adjustments of effect estimates and types of estimates on outcome were not presented as all results were derived from IPD analysis.

#### 4.2.1 Study type

All included studies were prospective cohort studies, namely the:

• Belgian Job Stress Project (BELSTRESS).
• Health and Social Support Study (HeSSup).
• Socio-Economic Panel (SOEP).
• National Child Development Study (NCDS).
• The prospective arm of the National Health and Nutrition Examination Survey (NHANES I).
• National Longitudinal Surveys of Young (NLSY79).
• American Change Life study (ACL).
• Alameda County Study.
• British Cohort Study (BCS).
• Midlife in the United States study (MIDUS).
• National Survey of Family and Households study (NSFH).
• Wisconsin Longitudinal Study (WLSG and WLSN).

We retrieved several publications of these studies focusing on several types of outcomes other than our outcomes of interest, or focusing on our outcomes of interest but using different exposure categories and/or types of outcome measures (Jensen et al., 2017, Molander et al., 2010, Herd et al., 2014, Holly and Mohnen, 2012, Hübler, 2019, Parent, 2000, Harford and Muthén, 2001, NHANES, 2010, Power and Elliott, 2006, Livingston et al., 2009, Virtanen et al., 2008, Moreau et al., 2004, Moreau et al., 2004, Bacquer et al., 2005). We included these studies after gaining access to individual participant data, which allowed us to conduct re-analyses aligned with our predefined exposure levels and outcome measures (see Section 3.6).

#### 4.2.2 Population studied

The included studies captured 110,043 workers (52,107 females and 57,937 males). All studies examined both female and male workers. The most commonly studied age groups were 30–44 years, followed by 45–59 years, ≥60 years and 15–29 years, respectively.

Most studies examined populations in the WHO regions of the Americas (eight studies from one country), followed by Europe (five studies from four countries) and the Western Pacific (one study from one country).

All studies included participants regardless of industrial sector, occupation and/or workplace setting.

#### 4.2.3 Exposure studied

Of the 14 included studies, ten measured exposure to long working hours using surveys, and four measured exposure by interviewing participants. Therefore, all studies relied on self-reported measures of working hours.

#### 4.2.4 Comparator studied

We re-analysed data of all included studies by applying 35–40 weekly work hours as the reference risk level.

#### 4.2.5 Outcomes studied

Eight studies measured alcohol consumption (in g/week) with surveys or interviews. Alcohol consumption was estimated by converting frequency and amount of drinks per week into g/week. This outcome was assessed through self-report in all studies assessing this outcome.

We could assess risky drinking in accordance to our pre-defined criteria in 12 studies.

Our searches did not find any study on the outcome of alcohol use disorder (prevalence, incidence or mortality).

### 4.3 Risk of bias within studies

#### 4.3.1 Risk of bias in studies assessing alcohol consumption in g/week

**Fig. 3:**
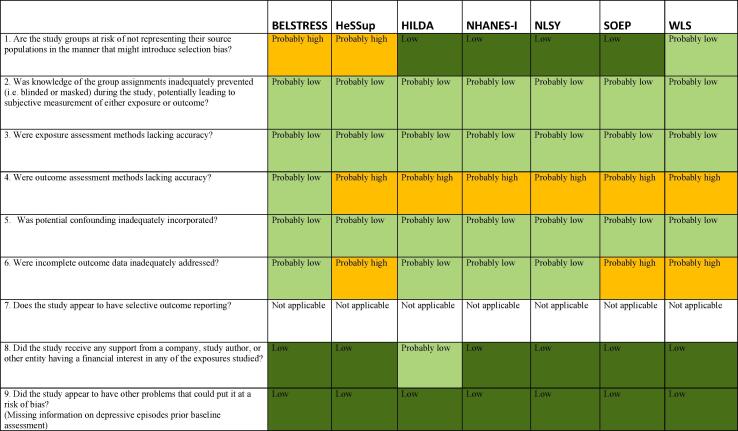
Summary of risk of bias across studies, Alcohol consumption (in g/week).

The risk of bias rating for each domain for all included studies for this outcome are presented in Fig. 3. The justification for each rating for each domain by included study is presented in 5, Berner et al., 2007, Health promotion, 1983 in the Supplementary data.

##### 4.3.1.1 Selection bias

Out of the seven studies that assessed alcohol consumption, four were considered low risk of bias, one was considered probably low risk of bias, and two were considered probably high risk of bias for selection bias, because their low response rates may have introduced bias.

##### 4.3.1.2 Performance bias

All studies were judged as probably low risk of bias for performance bias because we considered that not being blinded to the exposure (i.e. long working hours) would not have influenced the levels of alcohol consumption.

##### 4.3.1.3 Detection bias (exposure assessment)

All studies were considered probably low risk of bias for the domain of exposure assessment. All studies employed self-report to assess work hours, which has been proven to provide a reliable estimate.

##### 4.3.1.4 Detection bias (outcome assessment)

One study was judged as of probably low risk of bias, because the outcome was assessed using a standardized questionnaire. We judged the other studies to be probably high risk of bias, considering that no instrument for prospective record of alcohol consumption was applied, nor validated methods such AUDIT, and since the participants may have overestimated or underestimated their own alcohol use, for example due to social desirability bias.

##### 4.3.1.5 Confounding

Subgroup analyses were conducted considering the influence of sex and age on alcohol consumption. We therefore judged all studies as probably low risk of bias for this domain.

##### 4.3.1.6 Selection bias (incomplete outcome data)

We considered that attrition may have been related to both the exposure and the outcome. Participants exposed to long working hours may had been less available to follow-up surveys and interviews, as well as participants with increased alcohol consumption. Three studies were considered probably high risk of bias due to attrition rates or refusal rates as high as 20–40%. Four studies were judged as probably low risk of bias due to lower attrition rates, from 4.5 to 12%.

##### 4.3.1.7 Reporting bias

Since all included studies were unpublished studies for which we re-analysed data in accordance to our pre-defined exposure risk levels and outcome, we did not consider this domain in risk of bias assessment.

##### 4.3.1.8 Conflict of interest

We did not detect possible conflict of interests for any of included studies. One study was judged probably low risk of bias because we could not identify all funding sources.

##### 4.3.1.9 Other risk of bias

We did not identify any other source of bias in any of the included studies and considered all studies low risk of bias.

Overall, we judged the risk of bias to be probably high across studies assessing alcohol consumption in g/week, mainly because of predominance of use of non-validated instruments for measuring alcohol consumption and because of risk of bias for incomplete outcome data observed in some studies. None of the included studies was judged as low or probably low risk of bias for all domains (Fig. 3).

#### 4.3.2 Risk of bias in studies assessing risky drinking

**Fig. 4:**
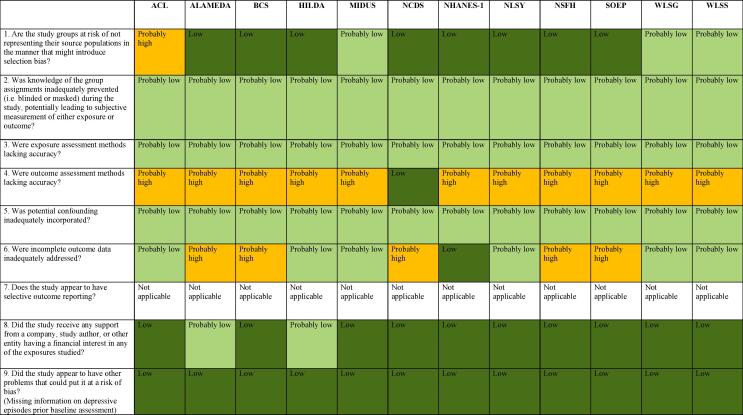
Summary of risk of bias across studies, Risky drinking.

Fig. 4 presents an overview of risk of bias in the studies that assessed risky drinking. Reasons for judgement are present in Appendix 5.

##### 4.3.2.1 Selection bias

The majority of studies assessing risky drinking were considered low risk of bias, due to the adequacy of sampling methods. Three studies were judged probably low risk of bias, due to response rates. One study was considered probably high risk of rate. In this study, probability sampling methods were employed. However, 30% of sampled households and 32% of sampled individuals were not interviewed.

##### 4.3.2.2 Performance bias

All studies were judged as probably low risk of bias for performance bias because we considered that not being blind to the exposure (i.e. long working hours) would not have influenced outcome assessment.

##### 4.3.2.3 Detection bias (exposure assessment)

All studies were considered probably low risk of bias for the domain of exposure assessment. The study employed self-report to assess work hours, which has been proved to provide a reliable estimate.

##### 4.3.2.4 Detection bias (outcome assessment)

Only one study assessed the outcome by using AUDIT, which is a validated instrument. The other studies were judged as probably high risk of bias, considering that no instrument for prospective record of alcohol consumption was applied, nor validated methods such AUDIT and that the participants may have overestimated or underestimated alcohol use.

##### 4.3.2.5 Confounding

Analyses were conducted by adjusting for sex, age and socioeconomic status. We therefore judged the study as probably low risk of bias for this domain.

##### 4.3.2.6 Selection bias (incomplete outcome data)

Risk of bias related to incomplete outcome data was appraised by considering attrition rates.

We judged five studies as probably high risk of bias for incomplete outcome data. Attrition rate in these studies ranged from 0.2 to 0.55. Six studies were considered probably low risk of bias for this domain and one was judged as low risk of bias.

##### 4.3.2.7 Reporting bias

Considering that all studies are unpublished studies for which we re-analysed data in accordance to our pre-defined exposure risk levels and outcome, we did not judge this domain of risk of bias assessment.

##### 4.3.2.8 Conflict of interest

The majority of studies were considered low risk of bias for conflict of interests. Funding sources of these studies were governmental agencies. Two studies reported multiple funding sources and were judged as probably low risk of bias.

##### 4.3.2.9 Other risk of bias

We did not identify any other source of bias.

Overall, we judged the risk of bias to be probably high across studies assessing risky drinking, mainly due to outcome assessment and incomplete outcome data (Fig. 4).

### 4.4 Synthesis of results

#### 4.4.1 Alcohol consumption in g/week

Seven studies with a total of 33,734 participants from three WHO regions reported estimates of the effect of exposure to long working hours on alcohol consumption (in g/week), compared with working standard hours (35–40 h/week). All studies could be included in a quantitative meta-analysis because we generated analysis from raw data using our pre-specified parameters.

**Fig. 5:**
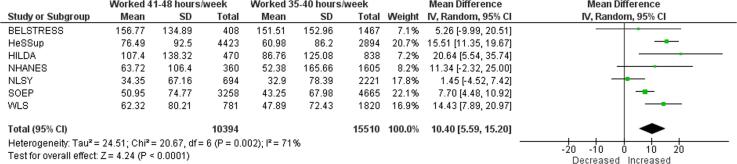
Main meta-analysis, Outcome: Alcohol consumption (in g/week), Comparison: 41–48 h/week compared with 35–40 h/week.

**Fig. 6:**
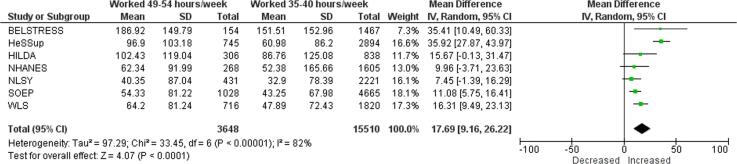
Main meta-analysis, Outcome: Alcohol consumption (in g/week), Comparison: 49–54 h/week compared with 35–40 h/week.

**Fig. 7:**
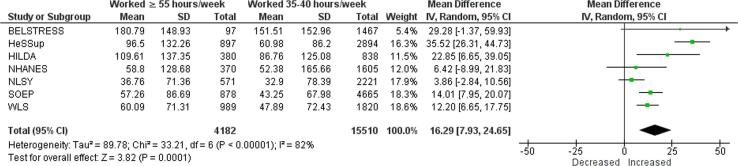
Main meta-analysis, Outcome: Alcohol consumption (in g/week), Comparison: ≥55 h/week compared with 35–40 h/week.

Compared with working 35–40 h/week, exposure to working 41–48 h/week increased consumption by 10.40 g/week (mean difference (MD); 95% confidence interval [CI] 5.59–15.20 g/week, 7 studies, 25,904 participants, I^2^ 71%; Fig. 5). Exposure to 49–54 work hours/week increased alcohol consumption in grams per week by 17.69 g/week (MD; 95% CI 9.16–26.22 g/week, 7 studies, 19,158 participants, I^2^ 82%; Fig. 6). Exposure to working ≥55 h/week increased alcohol consumption in grams per week by an estimated 16.29 g/week (MD; 95% CI 7.93–24.65 g/week, 4 studies, 19,692 participants, I^2^ 82%; Fig. 7).

#### 4.4.2 Risky drinking

**Fig. 8:**
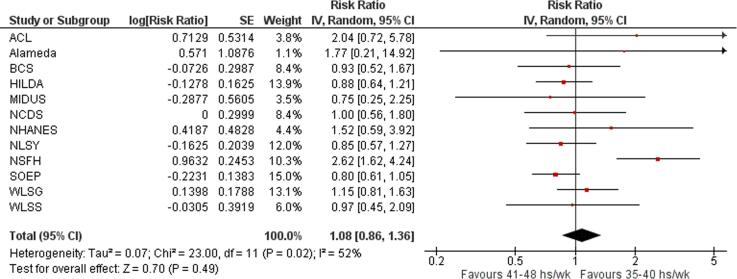
Forest plot, Risky drinking – 41–48 h/week.

**Fig. 9:**
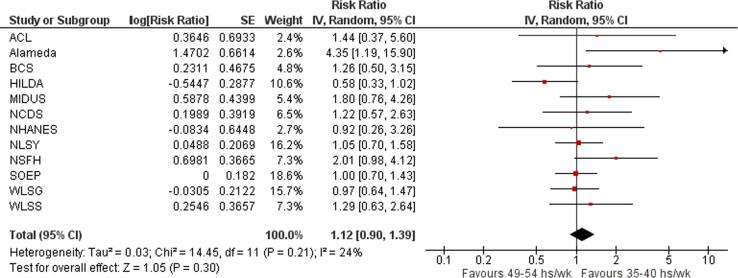
Forest plot, Risky drinking – 49–54 h/week.

**Fig. 10:**
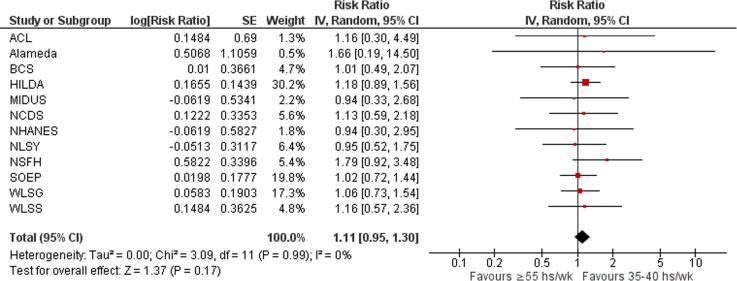
Forest plot, Risky drinking - *≥*55 h/week.

Twelve studies with a total of 4525 participants from three WHO regions provided estimates of the effect of exposure to long working hours on risky drinking, compared with working standard hours (35–40 h/week). Compared with working 35–40 h/week, exposure to working 41–48 h/week was estimated to have no effect on the risk of engaging in risky drinking (relative risk (RR) 1.08, 95% confidence interval [CI] 0.86–1.36, 12 studies, I^2^ 52%; Fig. 8), as well as exposure to working 49–54 h/week (RR 1.12, 95% CI 0.90–1.39, 12 studies, 3832 participants, I^2^ 24%; Fig. 9) or to ≥55 work hours/week (RR 1.11, 95% CI 0.95–1.30, 12 studies, 4525 participants, I^2^ 0%; Fig. 10).

#### 4.4.3 Additional analyses

To avoid issues related to multiple testing, we conducted subgroup analysis only for the comparison between ≥55 work hours/week and standard working hours. This comparison was chosen to investigate the effects of most extreme exposures to the risk factor.

#### 4.4.4 Alcohol consumption in g/week

##### 4.4.4.1 By WHO region

**Fig. 11:**
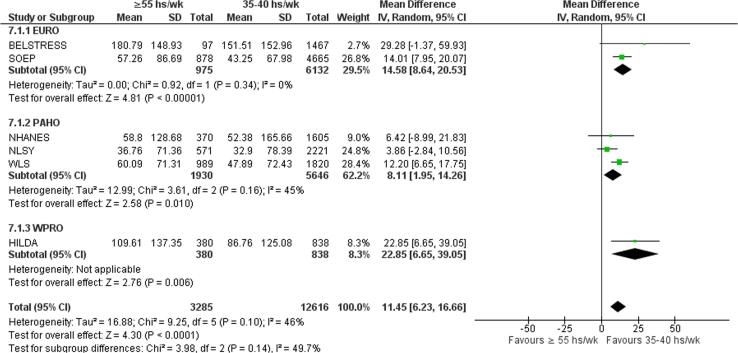
Subgroup analysis by WHO region, Alcohol consumption (g/wk).

Subgroup analysis by WHO region showed no subgroup differences (p = 0.14). Exposure to ≥55 work hours/week was associated with increase in alcohol consumption in all WHO regions for which data was included (Fig. 11).

##### 4.4.4.2 By sex

**Fig. 12:**
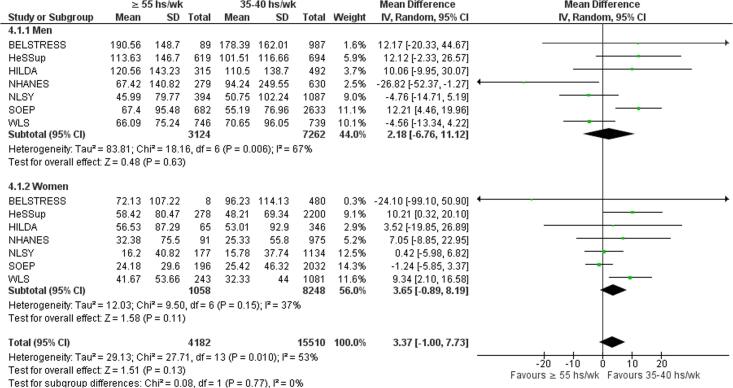
Subgroup analysis by sex, Alcohol consumption (g/wk).

Subgroup analysis by sex showed no statistically significant subgroup differences (p = 0.77) (Fig. 12).

##### 4.4.4.3 By age group

**Fig. 13:**
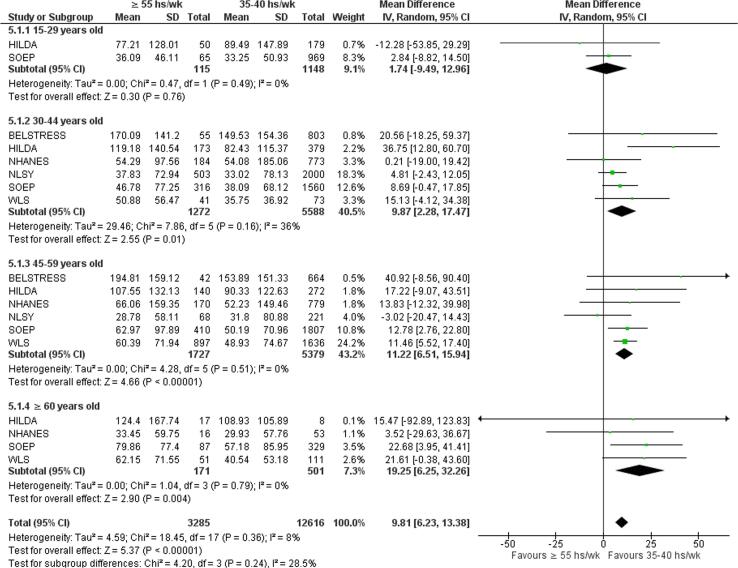
Subgroup analysis by age, alcohol consumption (g/wk).

Exposure to long working hours were associated with increased alcohol consumption for participants aged 30–44 years old (MD = 9.87, 95% CI 2.28–17.47, six studies, 6860 participants, I^2^ 36%); for participants aged 45–59 years old (MD = 11.22, 95% CI 6.51–15.94, six studies, 7106 participants, I^2^ 0%); and for participants aged ≥60 years old (MD = 19.25, 95% CI 6.25–32.26, four studies, 672 participants, I^2^ 28.5%). For patients aged 15–29 years old, there was no statistically significant difference between the exposure and control groups. Subgroup analysis by age group showed no statistically significant overall effect (p = 0.24) (Fig. 13).

##### 4.4.4.4 Subgroup analysis by socioeconomic status*,* alcohol consumption

**Fig. 14:**
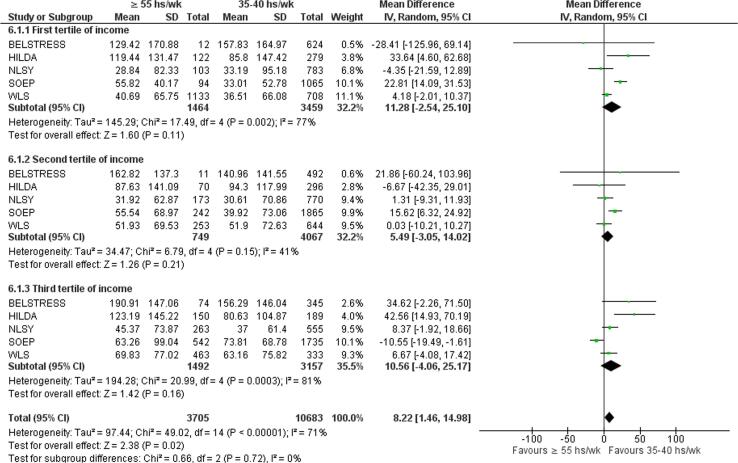
Subgroup analysis by socioeconomic status, alcohol consumption (g/wk).

Subgroup analysis for socioeconomic status as inferred by income showed a significant overall effect (p = 0.02), with no statistically significant subgroup differences (Fig. 14).

#### 4.4.5 Risky drinking

##### 4.4.5.1 By WHO region

**Fig. 15:**
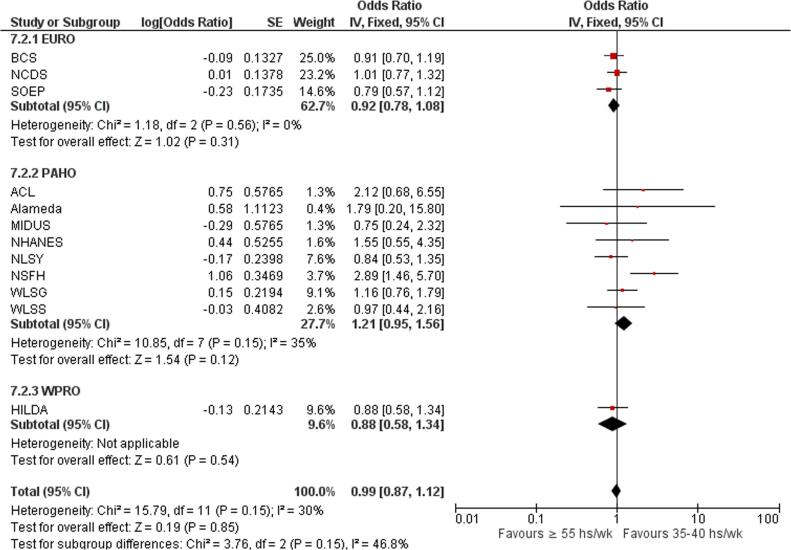
Subgroup analysis by WHO region, Risky drinking.

Subgroup analysis by WHO region did not show significant overall effects for any of the three regions with data included in the subgroup analysis; the test for subgroup differences was not statistically significant (Fig. 15).

##### 4.4.5.2 By sex

**Fig. 16:**
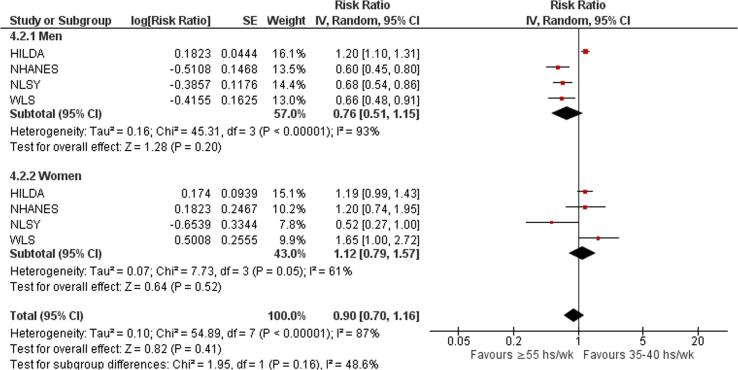
Subgroup analysis by sex, risky drinking.

Subgroup analysis by sex showed no statistically significant subgroup differences (p = 0.16) (Fig. 16).

##### 4.4.5.3 By age group

**Fig. 17:**
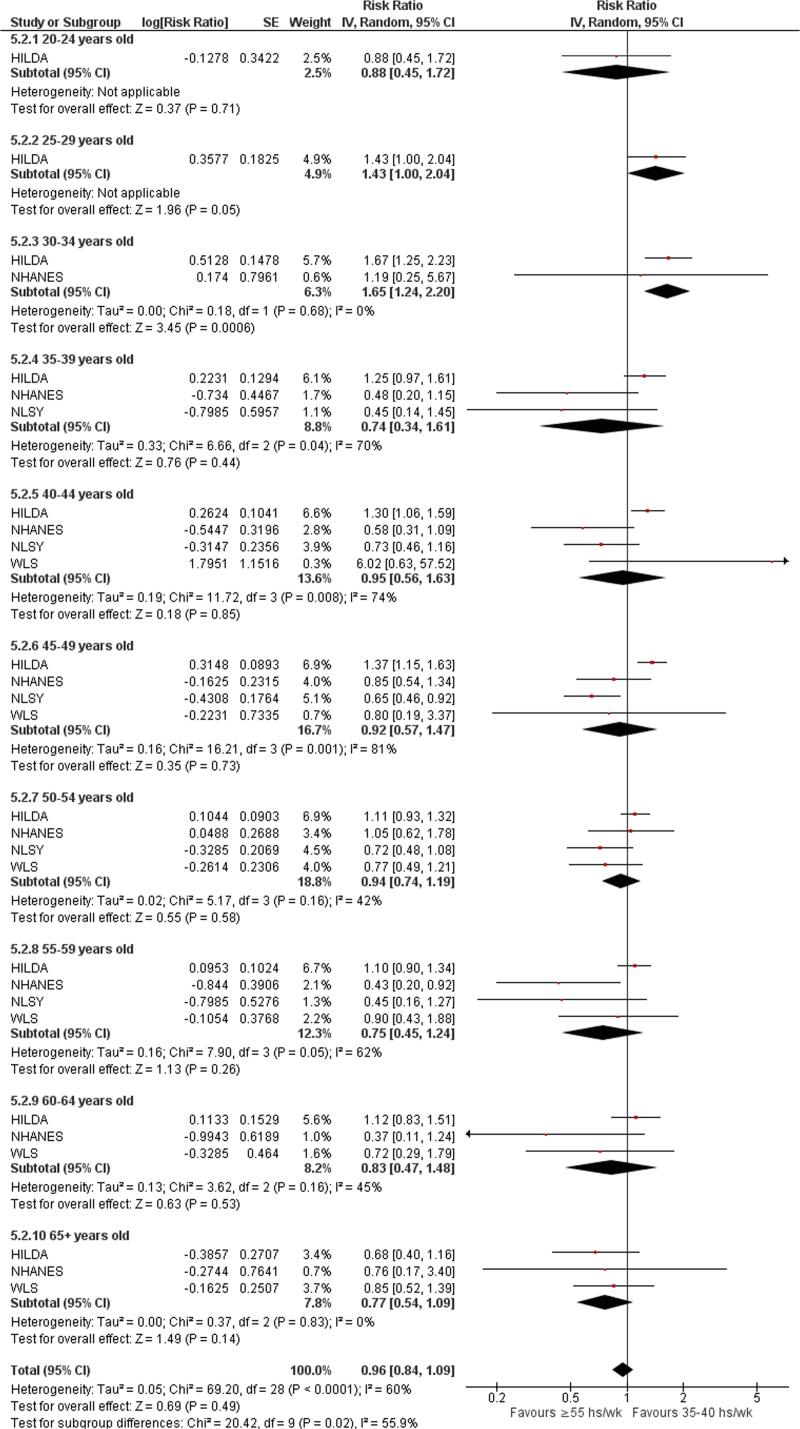
Subgroup analysis by age, Risky drinking.

Subgroup analysis by age group showed that the effect of the exposure of long working hours on risky drinking differs across age groups (p = 0.02). For participants aged 30–34 years old, exposure to long working hours increased the risk of risky drinking (RR = 1.65, 95% CI 1.24–2.20, two studies, I^2^ = 0%). This finding was not observed among other age groups (Fig. 17**)**.

### 4.5 Quality of evidence

#### 4.5.1 Alcohol consumption (in g/week)

Quality of evidence was appraised for each exposure level and the reference level of exposure. For all comparisons, quality of evidence was downgraded by two levels, due to risk of bias related to the use of non-validated tools for outcome measures and to the inconsistency of results across studies. When assessing inconsistency, we considered the I^2^ statistic, the p-value of the heterogeneity test, the direction of the effect of the exposure on the outcome and the overlap of confidence intervals. For the exposure to 41–48 h/week, the obtained value for the I^2^ statistic was 71%, with a p value for the heterogeneity test equal to 0.002. For both the exposures to 49–54 work hours/week and to ≥55 work hours/week, the I^2^ statistic was 82%, with a p value for the heterogeneity test lesser than to 0.001. For all comparisons, three studies showed no effect of the exposure to long working hours on alcohol consumption, and four studies showed increased alcohol consumption among participants exposed to long working hours.

#### 4.5.2 Risky drinking

**Fig. 18:**
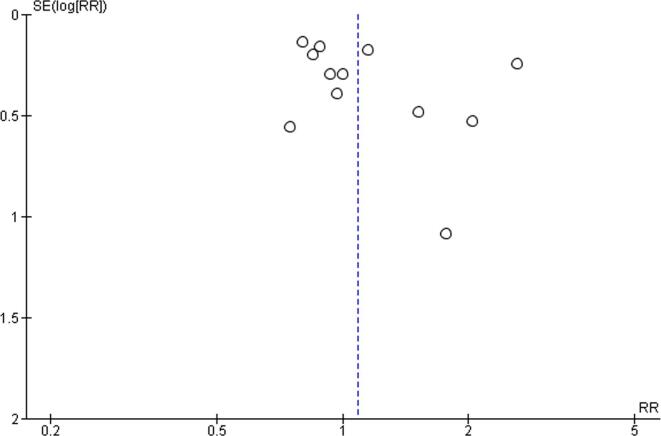
Funnel plot, Outcome: Risky drinking, Comparison: 41–48 h/week compared with 35–40 h/week.

**Fig. 19:**
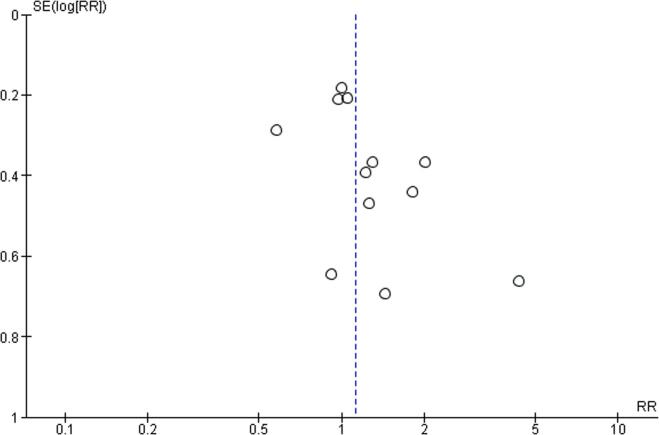
Funnel plot, Risky drinking, 49–54 h/week.

**Fig. 20:**
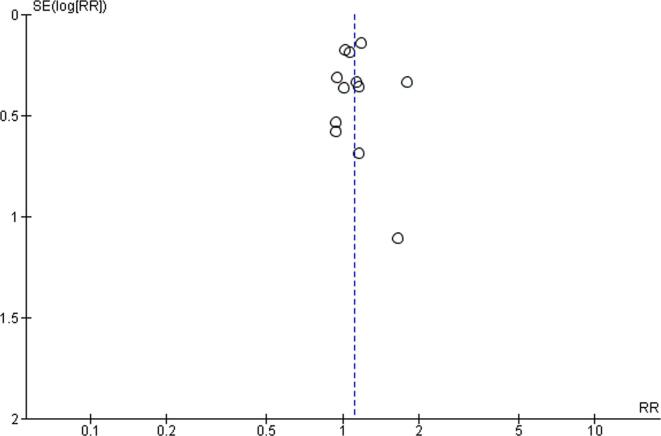
Funnel plot, Risky drinking, ≥55 h/week.

For all comparisons, quality of evidence was downgraded by at least one level, due to risk of bias related to the use of non-validated tools for outcome measures. For the exposure level of working 41–48 h/week, quality of evidence was additionally downgraded by one level due to inconsistency (I^2^ = 52%, p value for the heterogeneity test = 0.02), leading to the judgement of low quality. For the exposures to 49–54 work hours/week and to ≥55 work hours/week, quality of evidence was no further downgraded and considered moderate. Publication bias was explored by funnel plots, for all comparisons (Fig. 18, Fig. 19, Fig. 20). We did not downgrade quality of evidence for any of the comparisons due to publication bias.

### 4.6 Assessment of strength of evidence

According to our protocol we rated the strength of evidence based on a combination of four criteria outlined in the Navigation guide: (1) Quality of the entire body of evidence; (2) Direction of the effect estimate; (3) Confidence in the effect estimate; (4) Other compelling attributes.

#### 4.6.1 Quality of the entire body of evidence

Due to the low quality of the evidence for the majority of comparisons and outcomes and the lack of data on the effects of long working hours on the risk of acquiring alcohol use disorders, the body of evidence was considered not sufficient to assess the harmfulness of the exposure. In many studies, the methods for measuring alcohol consumption involved self-assessment by non-validated instruments, which may have introduced bias.

#### 4.6.2 Direction of the effect estimate

The study results were inconsistent in regards of the direction of the effect estimate across related outcomes. Exposure to long working hours increased alcohol consumption in all comparisons made but there were no statistically significant differences for risky drinking. This inconsistency prevents unequivocal conclusions on the effects of long working hours on alcohol consumption.

#### 4.6.3 Confidence in the effect estimate

The assumption of a dose–response relationship between the three exposure categories and alcohol consumption was not supported by our findings. Moreover, no intervention studies are available that demonstrate a reduction of the effect estimate as a consequence of reducing the exposure to minimal level.

#### 4.6.4 Other compelling attributes

We were not able to access data that could offer evidence for a discussion of other compelling attributes in assessing the strength of evidence. In summary, we conclude that there is inadequate evidence for harmfulness for all exposure categories for risky drinking and for exposure of 41–48 h/week and ≥55 h/week for alcohol consumption; and limited evidence for harmfulness for exposure to 49–54 h/week for increasing alcohol consumption.

## 5 Discussion

### 5.1 Summary of evidence

As shown in the table of summary of findings (Table 4), exposure to long working hours seems to be associated with an increased alcohol consumption in g/week for all risk levels of exposure. Exposure to working 41–48 h/week was associated with an increase of 10.4 g/week (CI 95% 5.59–15.20), based on low quality evidence (inadequate evidence of harmfulness). Exposure to 49–54 work hours/week was associated with an increase of 17.69 g/week (CI 95% 9.16–26.22), based on low quality evidence (inadequate evidence of harmfulness). Exposure to working ≥55 h/week may be associated to an increase of 16.29 g/week (CI 95% 7.93–24.65); this evidence was also of low quality (inadequate evidence of harmfulness). A dose–response gradient was not observed, which could be attributed to the imprecision of estimates or to a real absence of such gradient. It is conceivable that exposure to working ≥55 h/week may be associated with less available time for social activities and hence alcohol consumption.

Exposure to long working hours was not associated with the risk of risky drinking, for any of the comparisons. Exposure of working 41–48 h/week was associated with a relative risk of 1.08 (CI 95% 0.86–1.36), based on low quality evidence (inadequate evidence of harmfulness). Findings for the comparisons of 49–54 work hours/week and ≥55 work hours/week were based on moderate quality evidence and showed no differences in relation to standard weekly work hours, with relative risks of 1.12 (CI 95% 0.90–1.39) and 1.11 (0.95–1.30), respectively (inadequate evidence of harmfulness). Considering the findings for alcohol consumption in grams per week, these results suggest that exposure to long working hours may increase alcohol consumption, but not to an extent to increase the risk of acquiring risky drinking.

We did not find any study assessing the effects of the exposure to long working hours on alcohol use disorders, for which we could have permission to analyse and report data (see Table 6).

**Table 6:** Table of summary of findings.

	**Effect of exposure to long working hours on alcohol consumption, risky drinking and alcohol use disorder among workers**							
	**Population:** workers **Settings:** all countries and work settings **Exposure:** worked 41–48, 49–54 or ≥55 h/week **Comparison:** worked 35–40 work hours/week							
**Outcomes**	**Exposure category**	**Illustrative comparative risks (95% CI)**		**Relative effect (95% CI)**	**No. of participants (studies)**	**Quality of the evidence**	**Strength of evidence for human evidence**	**Comments**
		**Assumed risk** Unexposed workers	**Corresponding risk** Exposed worker					
**Alcohol consumption** (unit: g/week)	**Worked 41**–**48 h/week**	Not applicable	Not applicable	MD 10.40 g/week (5.59–15.20)	25,904 participants (7 studies)	⊕⊕⊝⊝^a, b^ Low	Inadequate evidence for harmfulness	5.59–15.20 more grams of alcohol consumed per week
	**Worked 49**–**54 h/week**	Not applicable	Not applicable	MD 17.69 g/week (9.16–26.22)	19,158 participants (7 studies)	⊕⊕⊝⊝^a, b^ Low	Inadequate evidence for harmfulness	9.16–26.22 more grams of alcohol consumed per week
	**Worked** ≥**55 h/week**	Not applicable	Not applicable	MD 16.29 g/week (7.93–24.65)	8794 participants (4 studies)	⊕⊕⊝⊝^a, b^ Low	Inadequate evidence for harmfulness	7.93–24.65 more grams of alcohol consumed per week
**Has engaged in risky drinking** (defined as: consumed >14 drinks/week for women and >21 drinks/week for men)	**Worked 41**–**48 h/week**	521 per 10,000	563 per 10,000 (448–709)	RR 1.08 (0.86–1.36)	6325 participants (12 studies)	⊕⊕⊝⊝^a, b^ Low	Inadequate evidence for harmfulness	73 fewer to 188 more per 10,000
	**Worked 49**–**54 h/week**	521 per 10,000	584 per 10,000 (469–724)	RR 1.12 (0.90–1.39)	3832 participants (12 studies)	⊕⊕⊕⊝^a^ Moderate	Inadequate evidence for harmfulness	52 fewer to 203 more per 10,000
	**Worked** ≥**55 h/week**	521 per 10,000	578 per 10,000 (495–677)	RR 1.11 (0.95–1.30)	4525 participants (12 studies)	⊕⊕⊕⊝^a^ Moderate	Inadequate evidence for harmfulness	26 fewer to 156 more per 10,000
**Has alcohol use disorder**	–	–	–	–	–	–	–	No evidence was found on this outcome.
**Acquired alcohol use disorder**	–	–	–	–	–	–	–	No evidence was found on this outcome.
**Died due to alcohol use disorder**	–	–	–	–	–	–	–	No evidence was found on this outcome.

**CI**: confidence interval; **MD**: mean difference; **RR**: risk ratio.

Navigation Guide quality of evidence ratings.

**High quality:** Further research is very unlikely to change our confidence in the estimate of effect.

**Moderate quality:** Further research is likely to have an important impact on our confidence in the estimate of effect and may change the estimate.

**Low quality:** Further research is very likely to have an important impact on our confidence in the estimate of effect and is likely to change the estimate.

^a^ Downgraded by one grade, because of serious risk of bias.

^b^ Downgraded by one grade, because of inconsistency.

^c^ Downgraded by one grade, because of imprecision.

### 5.2 Comparison to previous systematic review evidence

The effects of being exposed to long working hours on the risk for engaging in risky drinking was previously investigated in the 2015 Virtanen systematic review and meta-analysis. Based on two published and eighteen unpublished prospective studies, this systematic review found that long working hours were associated with a 12%increase of the odds of risky drinking (OR 1.12, CI 95% 1.04–1.20), however neither quality, nor strengths of this evidence were reported. The study by Marchand et al was the one with the greatest weight in meta-analysis. This study was not included in our systematic review, because the threshold for risky drinking was different from the one we adopted, namely ≥10 standard drinks/week for females and ≥15 standard drinks/week for males, and due to the lack of published results for analysis matching our risk levels of exposure. Attempts to contact corresponding authors to allow reanalysis of data were unsuccessful. We are not aware of any other systematic review or meta-analysis on the effect of exposure to long working hours on alcohol consumption (in g/week) or the risk of having, acquiring or dying due to alcohol use disorder, so there is no other systematic review or meta-analytic evidence against that we could compare our results on these outcomes.

### 5.3 Limitations and strengths of this systematic review

#### 5.3.1 Limitations of this review

Our systematic review was limited by the absence of published studies with our predefined exposure categories and at the same time also our eligible outcomes. All included studies are unpublished studies, for which we gained data access specifically for our IPD analyses. Attempts were made to contact authors of all retrieved primary studies that could provide data for our systematic review, but in several cases these attempts were not successful. We therefore acknowledge that several studies with potentially eligible data could not be included in our systematic review, and this has probably influenced the effect estimates, especially for the estimates based on low quality evidence. This absence of published studies also hampered our risk of bias assessment. Risk of bias assessment was performed based on the information retrieved on the study web sites or in related publications of the included studies, but we recognize that this information in some cases might not have been the most accurate to underpin our assessments.

#### 5.3.2 Strengths

Our systematic review and meta-analysis have several strengths, including adherence to all recommended steps of the Navigation Guide (Woodruff and Sutton, 2014), such as developing a protocol and assessing risk of bias, quality of evidence and strength of evidence, using Navigation Guide tools and approaches. Previous systematic reviews on the topic have not comprehensively provided detailed analyses across all analytic steps of the systematic review and meta-analysis.

Finally, to our knowledge, this is the first systematic review and meta-analysis conducted specifically for a global occupational burden of disease study, and as such it provides a model for future systematic reviews that will help ensure that these global health estimates adhere fully with GATHER (Stevens et al., 2016)

### 5.4 Use of evidence for burden of disease estimation

This systematic review and meta-analysis was conducted by WHO and ILO and supported by a large network of individual experts for the development of the WHO/ILO Joint Estimates (Ryder, 2017). More specifically, it sought to provide the necessary evidence base for these organizations to produce estimates of the burden of deaths and DALYs from alcohol use disorder attributable to exposure to long working hours. The systematic review did not include studies on the effect of exposure to long working hours on alcohol use disorders. It did find evidence for alcohol consumption (in g/week), which may be useful as an intermediary outcome, on the causal pathway between long working hours and alcohol use disorder. Producing estimates of the burden of alcohol use disorder attributable to exposure to long working hours is therefore however not sufficiently evidence-based (unless alcohol consumption can be used as an intermediary in some way) and therefore not warranted, and the parameters reviewed (including the pooled MDs from the meta-analyses for alcohol consumption) appear unsuitable as input data for WHO/ILO modelling of work-related burden of disease and injury, at this point.

## 6 Conclusions

There is low quality evidence indicating that long working hours may be associated with an increase in alcohol consumption. This finding was observed for all categories of risk levels, but without a dose–response gradient. There is low to moderate quality indicating that exposure to long working hours is not associated with risky drinking. This finding was observed for all categories of risk levels). Overall, we considered that there is inadequate evidence of harmfulness of long working hours on acquiring risky drinking at this time and that additional well-designed studies are still needed.

Subgroup analyses indicated that age may influence the association between long working hours and both alcohol consumption in gram per week and risky drinking. Alcohol consumption seems to be increased as an effect of the exposure of long working hours for the age group of 45–59 years old, and risky drinking for the age group of 30–34 years old. Subgroup analysis by socioeconomic status or WHO region did not identify subgroup effects. We did not find any study assessing the effects of the exposure to long working hours on alcohol use disorder, for which we could have permission to report data.

## 7 Differences between protocol and systematic review

• We did not consider risky drinking as an outcome during the protocol stage. However, considering the relevance and the availability of data for this outcome and the scarcity of data focusing on alcohol use disorder, this outcome was included at the review stage.
• We planned to perform sensitivity analyses and subgroup analysis that were not possible due to the homogeneity of studies in regards of risk of bias and the scarcity of studies providing data related to alcohol use disorder.
• We planned to contact experts with a list of included studies, with the request to identify potentially eligible additional studies. Due to the large number of references retrieved, we did not do this.

## Funding

All authors are salaried staff members of their respective institutions. The publication was prepared with financial support from the World Health Organization cooperative agreement with the Centres for Disease Control and Prevention National Institute for Occupational Safety and Health of the United States of America (Grant 1 E11 OH0010676-02).

## Sponsors

The sponsors of this systematic review are the World Health Organization and the International Labour Organization.

## Author contributions

Daniela Pachito took the following roles: Conceptualization, Formal analysis, Investigation, Methodology, Supervision, Validation, Visualization, Writing - original draft, Writing - review & editing.

Coordinated the entire series of systematic reviews: FP, YU.

Selected the lead reviewers and gathered the review teams: FP, YU.

Were the lead reviewers of this systematic review: DVP, LG.

Led the design of the systematic review including developed the standard methods: FP, YU.

Contributed substantially to the design of the systematic review: DVP, LG, EB, AD, ED, MCL, COCL, JL, ALCM, RR, RR, GS, JS.

Conducted the search: DVP, COCL.

Selected studies: DVP, LG, EB, ED, MCL, COCL, ALCM.

Extracted data: DVP, LG.

Requested missing data: DVP, LG.

Assessed risk of bias: DVP, LG, RR.

Coordinated risk of bias assessment across systematic reviews focusing on LWH: AD, RR, JL, GS, JS.

Conducted IPD analyses: LG, JB, EC, DB, KK, HG, SPP, LS, MS, SS, GV.

Conducted the meta-analyses: DVP, LG.

Assessed quality of evidence: DVP, LG, RR, GS.

Assessed evidence on causality: DVP, LG.

Developed the standards and wrote the template for all systematic reviews in the series: FP.

Wrote the first draft of the manuscript using the template: DVP, LG, MCL.

Revising the manuscript critically for important intellectual content: FP.

Ensured tailoring of the systematic review for WHO/ILO estimation purposes: FP.

Ensured harmonization across systematic reviews in the series: FP.

Approved the final version of the systematic review to be published: All authors.

Agreed to be accountable for all aspects of the work in ensuring that questions related to the accuracy or integrity of any part of the work are appropriately investigated and resolved: All authors.

## Declaration of Competing Interest

The authors declare that they have no known competing financial interests or personal relationships that could have appeared to influence the work reported in this paper.
