# Supplementary material for: The effect of exposure to long working hours on alcohol consumption, risky drinking and alcohol use disorder: A systematic review and meta-analysis from the WHO/ILO Joint Estimates of the Work-related burden of disease and injury
Source: Environ Int. 2021 Jan;146:106205. doi: 10.1016/j.envint.2020.106205 (PMC7786792; doi:10.1016/j.envint.2020.106205)
Supplement: Supplementary Data 1 [file mmc1.docx]

Appendix 1 Overview of the PECO Criteria

| Population | Working-age (≥ 15 years) workers in the formal and informal economy. |
| --- | --- |
| Exposure | Long working hours, defined as working hours > 40/week hours, i.e. working hours exceeding standard working hours that are defined as 35-40 hours/week |
| Comparison (reference level) | Standard working hours defined as working hours of 35-40 hours/week |
| Outcomes | - Absolute measures of alcohol consumption measured in grams of alcohol consumed per average week - Prevalence of, incidence of or mortality from any alcohol use disorder, as defined by the ICD-10 codes: F10, G72.1, Q86.0, X45 |
| Study designs | Randomized controlled trials (including parallel-group, cluster, cross-over and factorial trials), cohort studies (both prospective and retrospective), case-control studies, and other non-randomized intervention studies (including quasi-randomized controlled trials, controlled before-after studies and interrupted time series studies). |

Appendix 2 – Search strategies

| MEDLINE via Ovid | #1 Work Schedule Tolerance/  #2 Work-Life Balance/  #3 (work$ adj3 hour$).ti,ab,kw.  #4 (work$ adj3 schedul$).ti,ab,kw.  #5 overtime.ti,ab,kw.  #6 (work$ and (life$ or live$) and ($balance$ or interference$)).ti,ab,kw.  #7 (work$ and (famil$) and (conflict$)).ti,ab,kw.  #8 (work$ adj3 roster$).ti,ab,kw.  #9 (work organi?ation).ti,ab,kw.  #10 workweek$.ti,ab,kw.  #11 (work week$).ti,ab,kw.  #12 (work$ time$).ti,ab,kw.  #13 worktime$.ti,ab,kw.  #14 overwork$.ti,ab,kw.  #15 (work$ overload$).ti,ab,kw.  #16 (work$ adj3 extend$).ti,ab,kw.  #17 (work* adj3 compresse$).ti,ab,kw.  #18 OR/1-17  #19 Alcohol use disorder/  #20 Alcohol Drinking/  #21 Alcohol.ti,ab,kw.  #22 Drink$ Alcohol.tw.  #23 Alcohol consumption.tw.  #24 Alcohol dependence.tw.  #25 Alcoholic intoxication.tw.  #26 Alcohol addiction.tw.  #27 Alcohol abus$.tw.  #28 Binge drink$.tw.  #29 Alcoholism.tw.  #30 Alcohol misus$.tw.  #31 Substance abus$.tw.  #32 Problem$ drinking.tw.  #33 Drunkenness.tw.  #34 Fetal Alcohol Spectrum Disorders/  #35 fetal alcohol* syndrome.ti,ab.  #36 fetal alcohol* spectrum.ti,ab.  #37 alcohol* myopathy.ti,ab.  #38 poison* alcohol*.ti,ab.  #39 OR/19-38  #40 #18 AND #39  Hits returned: 935 |
| --- | --- |
| International Clinical Trials Register Platform | Work Schedule Tolerance OR Work-Life Balance OR Life-Work Imbalance OR work hour OR work schedule OR overtime OR work life balance OR work life interference OR work family conflict OR work roster OR work organization OR work week OR overwork OR work extend  Hits returned: 17 |
| Clinical Trials | (Work Schedule Tolerance OR Work-Life Balance OR Life-Work Imbalance OR work hour OR work schedule OR overtime OR work life balance OR work life interference OR work family conflict OR work roster OR work organization OR work week OR overwork OR work extend) AND alcohol OR alcoholism OR drinking  Hits returned: 42 |
| PubMed | #1 ("Work Schedule Tolerance"[Mesh])  #2 work schedule tolerance[Text word]  #3 work schedule tolerances[Text word]  #4 ("Work-Life Balance"[Mesh])  #5 work-life balance[Text word]  #6 work-life Imbalance[Text word]  #7 work-life harmony[Text word]  #8 work hour*[Text word]  #9 work schedul*[Text word]  #10 overtime [Title/Abstract]  #11 work life balance[Text Word]  #12 work live balance[Text Word]  #13 work life interference[Text Word]  #14 work live interference[Text Word]  #15 work* roster*[Text Word]  #16 work organization[Text Word]  #17 work organisation[Text Word]  #18 workweek* [Text Word]  #19 work week*[Text Word]  #20 work time*[Text Word]  #21 worktime [Text Word]  #22 overwork* [Text Word]  #23 work* overload*[Text Word]  #24 work extend*[Title/Abstract]  #25 work* compresse*[Title/Abstract]  #26 work* famil* conflict*[Title/Abstract]  #27 #1-26 OR  #28 "Alcoholism"[Mesh]  #29 alcohol addiction[Text Word]  #30 alcohol dependen*[Text Word]  #31 alcohol related disorder[Text Word]  #32 alcohol use disorder[Text Word]  #33 alcohol-induced disorder*[Text Word]  #34 alcohol-related disorder*[Text Word]  #35 alcoholic*[Title/Abstract]  #36 chronic alcoholism[Text Word]  #37 dipsomania[Text Word]  #38 ethanol dependen*[Text Word]  #39 problem* drink*[Text Word]  #40 drink* alcohol*[Title/Abstract]  #41 alcohol consumption[Title/Abstract]  #42 alcohol* intoxicat*[Title/Abstract]  #43 alcohol abus*[Title/Abstract]  #44 binge drink*[Title/Abstract]  #45 alcoholism[Title/Abstract]  #46 alcohol misus*[Title/Abstract]  #47 substance abus*[Title/Abstract]  #48 drunkenness[Title/Abstract]  #49 "Fetal Alcohol Spectrum Disorders"[Mesh]  #50 fetal alcohol* syndrome[Title/Abstract]  #51 fetal alcohol* spectrum[Title/Abstract]  #52 alcohol* myopathy[Title/Abstract]  #53 poison* alcohol*[Title/Abstract]  #54 #28-53 OR  #55 #27 AND #54  Hits returned: 689 |
| Embase via Ovid | #1 exp work schedule/  #2 work schedule tolerance.tw.  #3 work table.tw.  #4 exp work-life balance/  #5 work-life harmony.tw.  #6 work$ adj3 hour$.tw.  #7 work$ adj3 schedul$.tw.  #8 overtime.ab,ti.  #9 work life balance.tw.  #10 work life interference.tw.  #11 (work$ adj3 roster$).tw.  #12 (work adj3 organi$ation).tw.  #13 workweek$.ab,ti.  #14 work week$.ab,ti.  #15 work$ time$.ab,ti.  #16 worktime.tw.  #17 overwork$.ab,ti.  #18 work$ overload$.ab,ti.  #19 work$ adj3 extend$.ab,ti.  #20 work$ adj3 compress$.ab,ti.  #21 work$ famil$ conflict$.ab,ti.  #22 OR #1/21  #23 exp alcoholism/  #24 alcohol addiction.tw.  #25 alcohol dependen$.tw.  #26 alcohol use disorder$.tw.  #27 alcohol-induced disorder$.tw.  #28 alcohol-related disorder$.tw.  #29 alcoholic$.ab,ti.  #30 chronic alcoholism.tw.  #31 dipsomania.tw.  #32 ethanol dependen$.tw.  #33 problem$ drink$.tw.  #34 drink$ alcohol$.ab,ti.  #35 alcohol consumption.ab,ti.  #36 alcohol$ adj3 intoxicat$.ab,ti.  #37 alcohol abus$.ab,ti.  #38 binge drink$.ab,ti.  #39 alcoholism.ab,ti.  #40 alcohol misus$.ab,ti.  #41 substance abus$.ab,ti.  #42 drunkenness.ab,ti.  #43 exp fetal alcohol syndrome/  #44 f#tal alcohol$ syndrome.ab,ti.  #45 f#tal alcohol$ spectrum.ab,ti.  #46 alcohol$ myopathy.ab,ti.  #47 poison$ adj3 alcohol$.ab,ti.  #48 OR #23-47  #49 #22 AND #48  Hits returned: 692 |
| Web of Science | #1 TS=(Work Schedule Tolerance)  #2 TS=(Work-Life Balance)  #3 TS=(work NEAR hour)  #4 TS=(work NEAR schedule)  #5 TS=(overtime)  #6 TS=(work AND (life OR live) AND (balance OR interference))  #7 TS=(work AND family AND conflict)  #8 TS=(work NEAR roster)  #9 TS=(work organisation) OR TS=(work organization)  #10 TS=(workweek) OR TS=(work week)  #11 TI=(work time) OR TS=(worktime)  #12 TS=(overwork)  #13 TS=(work overload)  #14 TS=(work NEAR extend)  #15 TS=(work NEAR compressed)  #16 #1 OR #2 OR #3 OR #4 OR #5 OR #6 OR #7 OR #8 OR #9 OR #10 OR #11 OR #12 OR #13 OR #14 OR #15  #17 TS= (Alcohol use disorder)  #18 TS=(Alcohol Drinking)  #19 TI=(Alcohol)  #20 TS=(Drink Alcohol) OR TS=(Alcohol consumption) OR TS=(Alcohol dependence) OR TS=(Alcoholic intoxication) OR TS=(Alcohol addiction) OR TS=(Alcohol abuse)  #21 TS=(Binge drinking)  #22 TS=(Alcoholism) OR TS=(Alcohol misuse)  #23 TS=(Substance abuse) OR TS=(Problem drinking) OR TS=(Drunkenness)  #24 #17 OR #18 OR #19 OR #20 OR #21 OR #22 OR #23  #25 #16 AND #24  Hits returned: 6946 |
| CISDOC | Term used: Alcohol  Hits returned: 830 |
| PsycInfo | #1 MeSH: Work Schedule Tolerance  #2 MeSH: Work-Life Balance  #3 (Keywords: work schedule tolerance) OR (Keywords:work schedule tolerances) OR (Keywords: work-life balance) OR (Keywords: work-life Imbalance) OR(Keywords: work-life harmony) OR (Keywords: work hour) OR (Keywords: work schedul) OR (Keywords:work life balance) OR (Keywords: work live balance)OR (Keywords: work life interference) OR(Keywords: work live interference) OR (Keywords:work roster) OR (Keywords: work organization) OR(Keywords: work organisation) OR (Keywords:workweek) OR (Keywords: work week) OR(Keywords: work time) OR (Keywords: worktime) OR(Keywords: overwork) OR (Keywords: work overload)  #4 Title: overtime OR (Title: work extend) OR (Title: work compresse) OR (Title: work famil conflict) OR First Page: overtime OR (First Page: work extend) OR (First Page: work compresse) OR (First Page: work famil conflict)  #5 #1 OR #2 OR #3 OR #4  #6 MeSH: Alcoholism  #7 MeSH: Fetal Alcohol Spectrum Disorders  #8 (Keywords: alcohol addiction) OR (Keywords: alcohol dependent) OR (Keywords: alcohol related disorder) OR (Keywords: alcohol use disorder) OR (Keywords:alcohol-induced disorder) OR (Keywords: alcohol-related disorder) OR (Keywords: chronic alcoholism)OR Keywords: dipsomania OR (Keywords: ethanol dependent) OR (Keywords: problem drink)  #9 Title: alcoholic OR (Title: drink alcohol) OR (Title:alcohol consumption) OR (Title: alcohol intoxicat) OR(Title: alcohol abus) OR (Title: binge drink) OR Title:alcoholism OR (Title: alcohol misus) OR (Title:substance abus) OR Title: drunkenness OR (Title:fetal alcohol syndrome) OR (Title: fetal alcohol spectrum) OR (Title: alcohol myopathy) OR (Title:poison alcohol)  #10 First Page: alcoholic OR (First Page: drink alcohol)OR (First Page: alcohol consumption) OR (First Page: alcohol intoxicat) OR (First Page: alcohol abus)OR (First Page: binge drink) OR First Page:alcoholism OR (First Page: alcohol misus) OR (First Page: substance abus) OR First Page: drunkennessOR (First Page: fetal alcohol syndrome) OR (First Page: fetal alcohol spectrum) OR (First Page: alcohol myopathy) OR (First Page: poison alcohol)  #11 #6 OR #7 OR #8 OR #9 OR #10  #12 #5 AND #11  Hits returned: 15 |
| OpenGrey | #1 (Work Schedule Tolerance OR Schedule Tolerance Work OR Schedule Tolerances Work OR Tolerance Work Schedule OR Tolerances Work Schedule OR Work Schedule Tolerances OR Work-Life Balance OR Life-Work Imbalance OR work hour OR work schedule OR overtime OR work life balance OR work life interference OR work live balance OR work live interference OR work family conflict OR work roster OR work organization OR work organization OR workweek OR work week OR work time OR worktime OR overwork OR work overload OR work extend OR work compresse)  #2 (Alcohol-Related Disorders OR Alcohol use disorder OR Alcohol-Related Disorder OR Alcohol Drinking OR Alcohol Consumption OR Alcohol OR Drink Alcohol OR Alcohol consumption OR Alcohol dependence OR Alcoholic intoxication OR Alcohol addiction OR Alcohol abuse OR Binge drink OR Alcoholism OR Alcohol misuse OR Substance abuse OR Problem drinking OR Drunkenness)  #3 #1 AND #2  Hits returned: 71 |
| Grey Literature Report | #1 work  #2 Alcohol  #3 #1 AND #2  Hits returned: 39 |
| International Labour Organization | Term Used: Alcohol Drinking  Hits returned: 578 |
| World Health Organization | #1 (Work Schedule Tolerance OR Work-Life Balance OR Life-Work Imbalance OR work hour OR work life balance OR work life interference OR work organization OR work week OR worktime) #2 (Alcohol-Related Disorders OR Alcohol Drinking OR Alcohol Consumption)  #3 #1 AND #2  Hits returned: 64 |
| EUROSTAT | (Alcohol Consumption) AND (Work-Life Balance)  Hits returned: 219 |
| NIOSH | Term Used: Alcohol and work  Hits returned: 1196 |
| FIOH | Search term: Alcohol  Hits returned: 5 |
| Google AND Google Scholar | (Work Schedule Tolerance OR Work-Life Balance OR Life-Work Imbalance OR work hour OR work life balance OR work life interference OR work organization OR work week OR worktime)  AND  (Alcohol-Related Disorders OR Alcohol Drinking OR Alcohol Consumption)  Hits returned: 7040 |

Appendix 3 Description of missing data requested and received.

| **Study ID** | **Description of missing data** | **Person(s) from whom missing data were requested** | **Date of request(s)** | **Data or information**  **received** |
| --- | --- | --- | --- | --- |
| Marchand 2011 | Data related to exposure and outcome | Marchand A | 03/12/2018  05/01/2019 | No response |
| Bildt 2002 | Data related to exposure and outcome | Bildt C | 03/12/2018  05/01/2019 | No response |
| ACLS | Data related to exposure and outcome | House JS | 19/12/2018  05/02/2019 | No |
| ALAMEDA | Data related to exposure and outcome | Berkman L | 19/12/2018  05/02/2019 | No |
| BCS70 | Data related to exposure and outcome | Elliot J | 19/12/2018  05/02/2019 | No |
| Belstress | Data related to exposure and outcome | De Bacquer D | 19/12/2018 | Yes |
| DWECS | Data related to exposure and outcome | Feveile H | 19/12/2018  05/02/2019 | No |
| Vahtera 2002 | Data related to exposure and outcome | Vahtera J | 19/12/2018  05/02/2019 | No |
| HHS | Data related to exposure and outcome | Lahelma E | 19/12/2018 | Yes |
| HILDA | Data related to exposure and outcome | Summerfield M | 19/12/2018 | Yes |
| HeSSup | Data related to exposure and outcome | Korkeila K | 19/12/2018 | Yes |
| MIDUS | Data related to exposure and outcome | Bumpass L | 19/12/2018  05/02/2019 | No |
| NCDS | Data related to exposure and outcome | Power C | 19/12/2018 | Yes |
| NHANES | Data related to exposure and outcome | Madans JH | 19/12/2018  05/02/2019 | No |
| KLoSA | Data related to exposure and outcome | Cho S | 10/05/2019 | No |
| SOEP | Data related to exposure and outcome | Schupp J | 19/12/2018 | Yes |
| Suwazono 2003 | Data related to exposure and outcome | Suwazono Y | 10/05/2019  01/06/2019 | No |
| VATSPSUD | Data related to exposure and outcome | Bono R | 10/05/2019  01/06/2019 | No |

Appendix 4 Excluded studies and reason for their exclusion

| **Study (Study ID)** | **Reason for exclusion** |
| --- | --- |
| Bachman 2013 | Ineligible exposure studied |
| Baldwin 1999 | Ineligible study type |
| Baumert 2013 | Ineligible research question |
| Bazargan 2009 | Ineligible study type |
| Batista Ribeiro 2017 | Ineligible study type |
| Bono 2016 | Ineligible exposure studied |
| Breslin 2005 | Ineligible study type |
| Bromet 1990 | Ineligible study type |
| Bushnell 2010 | Ineligible outcome studied |
| Carriere 2005 | Ineligible exposure studied |
| Casson 1998 | Ineligible study type |
| Cheng 2012 | Ineligible study type |
| Copenhagen Male Study (unpublished study) | No access/permission to use data |
| Crum 1995 | Ineligible research question |
| Davey 2000 | Ineligible study type |
| Frone 1996 | Ineligible research question |
| Gibb 2012 | Ineligible exposure studied |
| Giousmpasoglou 2018 | Ineligible study type |
| Green 2016 | Ineligible research question |
| Hagihara 2001 | Ineligible study type |
| Holzgreve 2015 | Ineligible study type |
| Jang 2013 | Ineligible research question |
| Jaques 2015 | Ineligible study type |
| Jeon 2014 | Ineligible study type |
| Johnson 2004 | Ineligible exposure studied |
| Kaestner 2013 | Ineligible research question |
| Kawakami 1993 | Ineligible study type |
| Kawakami 1999 | Ineligible study type |
| Kim 2011 | Ineligible research question |
| Korean Longitudinal Study of Ageing (KLoSA) | Ineligible levels of exposure and outcome measures |
| Kouvonen 2008 | Ineligible research question |
| Lallukka 2010 | Ineligible study type |
| Lallukka 2008 | Ineligible study type |
| Laraqui 2011a | Ineligible research question |
| Laraqui 2011b | Ineligible research question |
| Lau 2017 | Ineligible research question |
| Macdonald 1999 | Ineligible study type |
| Malberg 2011 | Ineligible study type |
| Mansur 2015 | Ineligible research question |
| Marchand 2011 | Ineligible study type |
| Marchand 2008 | Ineligible study type |
| Marchand 2003 | Ineligible study type |
| McMorris 2000 | Ineligible exposure studied |
| Moore 2003 | Ineligible research question |
| Muntaner 1995 | Ineligible research question |
| Myers 2004 | Ineligible study type |
| Nakao 2006 | Ineligible research question |
| Nash 2010 | Ineligible study type |
| Nomoto 2015 | Ineligible study type |
| Ogasawara 2011 | Ineligible research question |
| Okechukwu 2015 | Ineligible study type |
| Oreskovich 2015 | Ineligible research question |
| Osilla 2015 | Ineligible exposure studied |
| Paschal 2004 | Ineligible exposure studied |
| Pham 2014 | Ineligible study type |
| Raymond 1962 | Ineligible research question |
| Renna 2008 | Ineligible research question |
| Roxburgh 1998 | Ineligible study type |
| Saeys 2014 | Ineligible study type |
| Saito 2004 | Ineligible study type |
| Schenker 1997 | Ineligible research question |
| Speicher 2010 | Ineligible study type |
| Suwazono 2003 | Ineligible research question |
| Taehyun 2016 | Ineligible research question |
| Takeda 1991 | Ineligible study type |
| Takeda 1992 | Ineligible study type |
| Trinkoff 1998 | Ineligible study type |
| Tsuboya 2015 | Ineligible study type |
| VATSPSUD (Bono 2016) | Ineligible levels of exposure and outcome measures |
| Virtanen 2015 | Ineligible study type |
| Virtanen 2012 | Ineligible research question |
| Wakai 2005 | Ineligible population studied |
| Weiss 1985 | Ineligible outcome studied |
| Yoo 2014 | Ineligible research question |
| 김명순 2004 | Ineligible study type |

Appendix 5 – Documentation of risk of bias assessment

# Study: HeSSup

Date of Assessment: May 16^th^ 2019

**References:**

Employment trajectory as determinant of change in health-related lifestyle: the prospective HeSSup study. Pekka Virtanen, Jussi Vahtera, Ulla Broms, Lauri Sillanmaki, Mika Kivimaki, Markku Koskenvuo. European Journal of Public Health, Vol. 18, No. 5, 504–508

| Domains | Low risk | Probably low risk | Probably high risk | High risk | Not applicable | Comment | Quote (indicate reference) |
| --- | --- | --- | --- | --- | --- | --- | --- |
| 1. Study group |  |  | X |  |  | Low response rate with the potential of introducing bias (e.g. people working long hours could be less available for answering the survey). | “The Time 1 postal  survey in 1998 yielded, with a response rate of 40.0%, 25 901  participants. (Reference 1)”. |
| 1. Blinding |  | X |  |  |  | Assessment of outcomes was made by the respondent himself/herself, but the fact of not having been blinded hardly influenced the obtained responses. | ‘The respondents reported their habitual frequency and amount of beer, wine and spirits consumed per week. This information was transformed into grams of absolute alcohol.’ |
| 1. Exposure assessment |  | X |  |  |  | Working hours were probably self-reported, which has been proved to provide precise estimates. | ‘The Time 1 postal survey in 1998 yielded, with a response rate of 40.0%, 25 901 participants. By the follow-up survey 5 years later (Time 2) 216  participants had died, 234 had moved abroad and 969 could not be reached due to unknown addresses.’ |
| 1. Outcome assessment |  |  | X |  |  | No instrument for prospective record of alcohol consumption was applied, nor validated methods such AUDIT. Participants may have overestimated or underestimated alcohol use. | “The respondents reported their habitual frequency and amount of beer, wine and spirits consumed per week. This information was transformed into grams of absolute alcohol.” |
| 1. Confounding |  | X |  |  |  | Analysis were adjusted to most important confounders (age, sex), but other confounders may also be relevant. |  |
| 1. Incomplete outcome data |  |  | X |  |  | 20% of attrition rate.  Reasons for missing outcome data potentially may have been related to outcome or unbalanced between exposed and unexposed groups. |  |
| 1. Selective outcome reporting |  |  |  |  | X | Unpublished study. |  |
| 1. Conflict of interest | X |  |  |  |  | Funding source was limited to government. | ‘J.V. and M.K. were supported by the Academy of Finland (grants 117604, 124271 and 124322).’  ‘Conflicts of interest: None declared.’ |
| 1. Other bias | X |  |  |  |  | We did not identify any other source of bias |  |

# Study: Belgium Job Stress (BELSTRESS)

Date of Assessment: May 16^th^, 2019

**References:**

#1 Perceived Job Stress and Incidence of Coronary Events: 3-Year Follow-up of the Belgian Job Stress Project Cohort. Dirk De Bacquer, E. Pelfrene, E. Clays, R. Mak, M. Moreau, P. de Smet, M. Kornitzer, and G. De Backer. Am J Epidemiol 2005;161:434–441

#2 Occupational stress and incidence of sick leave in the Belgian workforce: the Belstress study. M Moreau, F Valente, R Mak, E Pelfrene, P de Smet, G De Backer, M Kornitzer J Epidemiol Community Health 2004;58:507–516.

#3 Obesity, body fat distribution and incidence of sick leave in the Belgian workforce: the Belstress study. M Moreau, F Valente, R Mak, E Pelfrene, P de Smet, G De Backer2 and M Kornitzer. International Journal of Obesity (2004) 28, 574–582

| Domains | Low risk | Probably low risk | Probably high risk | High risk | Not applicable | Comment | Quote (indicate reference) |
| --- | --- | --- | --- | --- | --- | --- | --- |
| 1. Study group |  |  | X |  |  | Participants were recruited only from large industries or administration. Self-employed workers, workers of small to medium size companies were not included and may represent a different group in terms of developing the outcomes of interest in association with the exposure.  Methods for enrollment involved invitation letters, and the participation rate was low (48%). Participants that accepted to take part in the study may not be representative of the worker population. | ‘The sample consists of middle-aged men and women at work in 25 large industries or administrations across  Belgium. Among these were 13 large industrial plants, six bank and insurance companies, four public administrations,  and two hospitals willing to participate in the study’.  ‘All 44,530 employees aged 35–59 years received a personal invitation letter that included a reply form and a return envelope.’ |
| 1. Blinding |  | X |  |  |  | Assessment of outcomes was made by the respondent himself/herself, but the fact of not having been blinded hardly influenced the obtained responses. | ‘The self-administered  questionnaires were then distributed among the  interested participants together with a personal invitation for  the medical screening at the workplace. Participants were asked to complete the questionnaires at home and bring them  to the medical examination, which took place at least a week  later.’ |
| 1. Exposure assessment |  | X |  |  |  | Working hours were probably self-reported, which has been proved to provide precise estimates. | ‘Apart from work-related characteristics, the self-administered questionnaires were targeting information on  sociodemographic characteristics, lifestyle-related factors (smoking, alcohol consumption, dietary habits, physical  activity), medical history (diabetes, coronary heart disease, respiratory problems, family medical history), health perception,  and psychosocial scales for depression and anxiety’ |
| 1. Outcome assessment |  | X |  |  |  | A standardized questionnaire was used to measure alcohol consumption. | ‘Smoking habits and alcohol consumption: the standardised  questionnaire from the MONICA study was used’ (Ref #2) |
| 1. Confounding |  | X |  |  |  | Analysis were adjusted to most important confounders (age, sex), but other confounders may also be relevant. |  |
| 1. Incomplete outcome data |  | X |  |  |  | Low attrition rate (4.5%) (Based on Ref #2) |  |
| 1. Selective outcome reporting |  |  |  |  | X | Unpublished study. |  |
| 1. Conflict of interest | X |  |  |  |  | Funding sources were limited to governmental agencies. | ‘BELSTRESS was supported by grants from the Federal  Office for Scientific, Technical, and Cultural Affairs (ST/02/007), the FWO-Vlaanderen, and the National Fund for  Scientific Research (FNRS).’ |
| 1. Other bias | X |  |  |  |  | We did not identify any other source of bias |  |

# Study: NCDS - National Child Development Study

Date of Assessment: May 16^th^ , 2019

**References:**

#1 <https://ncds.info/>

#2 Cohort profile: 1958 British birth cohort (National Child Development Study). Chris Power and Jane Elliott. International Journal of Epidemiology 2006;35:34–41.

| Domains | Low risk | Probably low risk | Probably high risk | High risk | Not applicable | Comment | Quote (indicate reference) |
| --- | --- | --- | --- | --- | --- | --- | --- |
| 1. Study group | X |  |  |  |  | Inclusion criteria was date or birth. | ‘Participants are survivors from an original sample of over 17 000 births, all born in England, Wales, and Scotland, during 1 week in  1958, and followed-up by parental interview and examination at ages 7, 11, and 16 yr and by cohort member interview at 23, 33, and 42 yr.’ |
| 1. Blinding |  | X |  |  |  | Assessment of outcomes was made by the respondent himself/herself, but the fact of not having been blinded hardly could have influenced the obtained responses. | ‘Followed into adult life, the cohort had reached a life stage marked by major transitions—for example from school or fulltime  further education to employment (although unemployment  was very high), and from dependent status in their family of origin to independent status as heads of new households.  A survey at age 23 (1981) was designed to trace these transitions, and in so doing it differed from earlier follow-ups in obtaining information directly from the cohort member (instead of their parents, usually the mother)’ |
| 1. Exposure assessment |  | X |  |  |  | Working hours were self-reported, which has been proved to provide precise estimates. | ‘Followed into adult life, the cohort had reached a life stage marked by major transitions—for example from school or fulltime  further education to employment (although unemployment  was very high), and from dependent status in their family of origin to independent status as heads of new households.  A survey at age 23 (1981) was designed to trace these transitions, and in so doing it differed from earlier follow-ups in obtaining information directly from the cohort member (instead of their parents, usually the mother)’ |
| 1. Outcome assessment | X |  |  |  |  | Alcohol consumption was assessed with Audit | Table 2 |
| 1. Confounding |  | X |  |  |  | Analysis were adjusted to most important confounders (age, sex), but other confounders may also be relevant. |  |
| 1. Incomplete outcome data |  |  | X |  |  | Considering follow up from 23 years old to 42 years old, follow up loss was 2,5%. However, refusal rates were: 7.1% (23 yr); 11.1% (33 yr); and 13.2% (42yr). Refusal to collaborate may be potentially related to the exposure and outcome, therefore introducing bias. | ‘The main reasons for  sample loss over time are individuals moving to a new address and not responding to efforts to trace them. Refusal rates  are relatively low but also contribute to sample loss over time. At age 23 yr refusal was 7.1%; at 33 yr, 11.1%; and at 42 yr, 13.2%.’ |
| 1. Selective outcome reporting |  |  |  |  | X | Unpublished study. |  |
| 1. Conflict of interest | X |  |  |  |  | Funding source was limited to government. | ‘Research at the Institute of Child Health and the Great Ormond Street Hospital for Children NHS Trust benefits from R&D funding received from the NHS Executive. The Centre for Longitudinal Studies, with responsibility for the NCDS is funded  as a Resource Centre by the ESRC’. |
| 1. Other bias | X |  |  |  |  | We did not identify any other source of bias |  |

# Study: SOEP

Date of Assessment: 19/05/2019

**References:**

SOEP report # 1024/2019; SOEP report # 465/2012; SOEP core; report 477 Series C Data Documentation

| Domains | Low risk | Probably low risk | Probably high risk | High risk | Not applicable | Comment | Quote (indicate reference) |
| --- | --- | --- | --- | --- | --- | --- | --- |
| 1. Study group | X |  |  |  |  | Representative stratified sample. | ‘We used stratified sampling of sample points in order to assure minimum sample sizes for different regions of Germany’ (Text extracted rom report 477 Series C Data Documentation). |
| 1. Blinding |  | X |  |  |  | Assessment of outcomes was made by the respondent himself/herself, but the fact of not having been blinded hardly influenced the obtained responses. |  |
| 1. Exposure assessment |  | X |  |  |  | Working hours were self-reported, which has been proved to provide precise estimates. |  |
| 1. Outcome assessment |  |  | X |  |  | Self-reported survey |  |
| 1. Confounding |  | X |  |  |  | Analysis were adjusted to most important confounders (age, sex), but other confounders may also be relevant. |  |
| 1. Incomplete outcome data |  |  | X |  |  | Attrition rate related to the 10 yrs follow up of the first wage is around 0.55 to 0.6. (This is not specific for alcohol consumption) | SOEP-Core  Fig. 22 |
| 1. Selective outcome reporting |  |  |  |  | X | Unpublished study. |  |
| 1. Conflict of interest | X |  |  |  |  | Funding sources from Government. | From 1990 to 2002, SOEP was funded through the German National Science Foundation (Deutsche Forschungsgemeinschaft, DFG), partly supported by the Federal Ministry of Education and Research. As a Service Unit of the Leibniz Association, SOEP now receives continued funding through the Joint Science Conference (GWK, former Bund-Länder Commission for Educational Planning and Research Promotion) by the Federal Ministry of Education and Research (BMBF) and the State of Berlin. |
| 1. Other bias | X |  |  |  |  | We did not identify any other source of bias |  |

# Study: National Longitudinal Survey of Youth (NLSY)

Date of Assessment: June 8^th^ 2019

**References:**

<https://www.nlsinfo.org/content/cohorts/nlsy79>

Ref #1 Industry-Specific Capital and the Wage Profile: Evidence from the National Longitudinal Survey of Youth and the Panel Study of Income Dynamics. Daniel Parent. Journal of Labor Economics, 2000, 18 (2): 306-323.

Ref#2 The Dimensionality of Alcohol Abuse and Dependence: A Multivariate Analysis of DSM-IV Symptom Items in the National Longitudinal Survey of Youth. Thomas C. Harford, Bengt O. Muthén. J. Stud. Alcohol 62: 150-157, 2001

| Domains | Low risk | Probably low risk | Probably high risk | High risk | Not applicable | Comment | Quote (indicate reference) |
| --- | --- | --- | --- | --- | --- | --- | --- |
| 1. Study group | X |  |  |  |  | Nationally representative sample. | ‘In 1978 a list of housing units in selected areas of the United States was created for the first NLSY79 interview. Interviewers went to a random sample of these homes and performed a short interview, called the screener, which provided basic information on every resident of the household. Also using a random sample of Department of Defense records, the survey included members of the military.  Together these two processes provided information, such as name, age, sex, race, and address, on more than 155,000 people. This information was used to identify all individuals ages 14 to 21 as of December 31, 1978. Based on this screener information, each appropriately aged individual was assigned to one of the sample groups. Then, in 1979, interviewers asked individuals on this list to participate in the first NLSY79 interview. Any person who completed the first round interview is considered a member of the NLSY79 cohort.’  From the study web site. |
| 1. Blinding |  | X |  |  |  | Assessment of outcomes was made by the respondent himself/herself, but the fact of not having been blinded hardly could have influenced the obtained responses. |  |
| 1. Exposure assessment |  | X |  |  |  | Working hours were self-reported, which has been proved to provide precise estimates. |  |
| 1. Outcome assessment |  |  | X |  |  | No instrument for prospective record of alcohol consumption was applied, nor validated methods such AUDIT. Participants may have overestimated or underestimated alcohol use. | ‘the mean number of drinking days (frequency) was 7.3 (7.3) and the mean average number of drinks (average quantity) was 2.9’  From ref#2 |
| 1. Confounding |  | X |  |  |  | Analysis were adjusted to most important confounders (age, sex), but other confounders may also be relevant. |  |
| 1. Incomplete outcome data |  | X |  |  |  | Responses were obtained for over 90% of the sample. | ‘Of the total sample of current drinkers (N = 6,648), 5,984 provided complete information on each of the variables described below.’  From ref#2 |
| 1. Selective outcome reporting |  |  |  |  | X | Unpublished study. |  |
| 1. Conflict of interest | X |  |  |  |  | Study conducted by the Bureau of Labor Statistics |  |
| 1. Other bias | X |  |  |  |  | We did not identify any other source of bias |  |

# Study: HILDA

Date of Assessment: June 8^th^ 2019

**References:**

<https://melbourneinstitute.unimelb.edu.au/hilda>

Ref #1 Effects of Alcohol Consumption in Spousal Relationships. Michael Livingston. J Stud Alcohol Drugs. 2009 May;70(3):383-90.

Ref #2 HILDA Research Paper No. 35 – May 2004

| Domains | Low risk | Probably low risk | Probably high risk | High risk | Not applicable | Comment | Quote (indicate reference) |
| --- | --- | --- | --- | --- | --- | --- | --- |
| 1. Study group | X |  |  |  |  |  | ‘Households were selected using a multistage sampling approach, with areas (consisting of around 200 households) selected at random and then a number of households selected within each area.’  From Ref# 1 |
| 1. Blinding |  | X |  |  |  | Assessment of outcomes was made by the respondent himself/herself, but the fact of not having been blinded hardly influenced the obtained responses. |  |
| 1. Exposure assessment |  | X |  |  |  | Working hours were self-reported, which has been proved to provide precise estimates. | ‘Respondents in paid employment were asked to indicate the number of hours/week they usually work in all their jobs (including paid or unpaid overtime)’  From Ref #2 |
| 1. Outcome assessment |  |  | X |  |  | No instrument for prospective record of alcohol consumption was applied, nor validated methods such AUDIT. Participants may have overestimated or underestimated alcohol use. | ‘Alcohol consumption was measured using the standard quantity-frequency questions. For this measure, respondents answer two questions—how often they drink alcohol and how many standard drinks they have when they drink. The responses of these questions are then converted into estimates of average weekly consumption by converting the responses of these questions into the number of drinking occasions per year and the average amount consumed at each drinking occasion. This method of estimating alcohol consumption has been shown to underestimate actual consumption (e.g., Gmel et al., 2006; Stockwell et al., 2004), but it still produces estimates of respondents’ drinking levels in the correct rank order (Poikolainen et al., 2002), which is all that was required for the current study.’  From Ref# 1 |
| 1. Confounding |  | X |  |  |  | Analysis were adjusted to most important confounders (age, sex), but other confounders may also be relevant. |  |
| 1. Incomplete outcome data |  | X |  |  |  | Low attrition rate. |  |
| 1. Selective outcome reporting |  |  |  |  | X | Unpublished study. |  |
| 1. Conflict of interest |  | X |  |  |  | Multiple sources of funding were mentioned, but it is unlikely that this may have introduced bias. | ‘This program is funded through grants and research contracts provided by a variety of sources, including the ARC.’ |
| 1. Other bias | X |  |  |  |  | We did not identify any other source of bias |  |

# Study: WLS Wisconsin Longitudinal Study

Date of Assessment: June 8^th^ 2019

**References:**

<https://www.ssc.wisc.edu/wlsresearch/>

Ref #1 Age-Related Changes in Drinking Patterns From Mid- to Older Age: Results From the Wisconsin Longitudinal Study. Rachel C. Molander, James A. Yonker, and Dean D. Krahn. Alcoholism: Clinical and Experimental Research 2010; 34(7): 1182-1192

Ref #2 Cohort Profile: Wisconsin longitudinal study (WLS). Pamela Herd, Deborah Carr and Carol Roan. International Journal of Epidemiology 2014;43:34–41. doi:10.1093/ije/dys194

| Domains | Low risk | Probably low risk | Probably high risk | High risk | Not applicable | Comment | Quote (indicate reference) |
| --- | --- | --- | --- | --- | --- | --- | --- |
| 1. Study group |  | X |  |  |  | Inclusion criterion was year of graduation.  Rate of response for alcohol behaviors section was around 80%. | ‘WLS sample was originally comprised of over 10,000 men and women who graduated from Wisconsin high schools in 1957.’  ‘The WLS has enjoyed excellent response rates. In 1993, 8,493 completed the telephone interview (94% completion rate among living respondents who could be located). The alcohol behaviors section of the interview was randomly subsampled at just under 80%, and participants who completed this section constituted the baseline sample of 6,489.’  From Ref #1 |
| 1. Blinding |  | X |  |  |  | Assessment of outcomes was made by the responses of the participants to an interviewer. The fact of having been blinded hardly influenced the obtained responses. |  |
| 1. Exposure assessment |  | X |  |  |  | Working hours were self-reported, which has been proved to provide precise estimates. | ‘GR - Total number of hours graduate worked last week at all jobs. ^83^('Health promotion: Alcohol and drug misuse prevention,')^838383828181807978777675^[75]^75747474747474747473737373737373727170696969696968^ \|mode=phone\| (20b)’  From study code book. |
| 1. Outcome assessment |  |  | X |  |  | Instruments for prospective record of alcohol consumption were not applied, nor validated methods such AUDIT. Participants may have overestimated or underestimated alcohol use. | ‘During both survey waves (telephone interview) respondents were asked a series of alcohol-related questions that included (a) number of drinking days in the past month, (b) average number of drinks per drinking day in the past month, (c) number of binge drinking episodes (‡5 drinks) in the past month, and (d) lifetime history of drinking-related problems. Responses to (a) and (b) were multiplied to construct a measure of total alcohol consumption in the past month.’  From Ref #1 |
| 1. Confounding |  | X |  |  |  | Analysis were adjusted to most important confounders (age, sex, SES), but other confounders may also be relevant. |  |
| 1. Incomplete outcome data |  |  | X |  |  | Attrition rate near 30% |  |
| 1. Selective outcome reporting |  |  |  |  | X | Unpublished study. |  |
| 1. Conflict of interest | X |  |  |  |  | No conflict of interest detected. | ‘Since 1991, the WLS has been supported principally  by the National Institutes for Health, National  Institute on Aging, with additional support from the Vilas Estate Trust, the National Science Foundation, the Spencer Foundation, and the Grad’  From Ref #2 |
| 1. Other bias | X |  |  |  |  | No additional source of bias was identified. |  |

# Study: NAHNES I

Date of Assessment: May 18th, 2019

**References:**

Zipf G, Chiappa M, Porter KS, et al. National Health and Nutrition Examination Survey: Plan and operations, 1999–2010. National Center for Health Statistics. Vital Health Stat 1(56). 2013.

| Domains | Low risk | Probably low risk | Probably high risk | High risk | Not applicable | Comment | Quote (indicate reference) |
| --- | --- | --- | --- | --- | --- | --- | --- |
| 1. Study group | X |  |  |  |  | Representative sample | ‘NHANES was designed to assess the health and nutritional status of the civilian noninstitutionalized U.S. population. NHANES data were not obtained using a simple random sample. Rather, a complex, multistage probability sampling design was used to select a sample representative of the civilian noninstitutionalized household population of the United States.’ |
| 1. Blinding |  | X |  |  |  | Assessment of outcomes was made by the responses of the participants to an interviewer. The fact of having been blinded hardly influenced the obtained responses. |  |
| 1. Exposure assessment |  | X |  |  |  | Working hours were self-reported, which has been proved to provide precise estimates. |  |
| 1. Outcome assessment |  |  | X |  |  | Instruments for prospective record of alcohol consumption were not applied, nor validated methods such AUDIT. Participants may have overestimated or underestimated alcohol use. |  |
| 1. Confounding |  | X |  |  |  | Analysis were adjusted to most important confounders (age, sex), but other confounders may also be relevant. |  |
| 1. Incomplete outcome data | X |  |  |  |  | Attrition rate of 12%. |  |
| 1. Selective outcome reporting |  |  |  |  | X | Unpublished study. |  |
| 1. Conflict of interest | X |  |  |  |  | No conflict of interest detected. |  |
| 1. Other bias | X |  |  |  |  | No additional source of bias was identified. |  |

# Study: ACL

Date of Assessment: October 7^th^ 2019

Socioeconomic disparities in health change in a longitudinal study of US adults: the role of health-risk behaviors. Lantz et al. Social Science & Medicine 53 (2001) 29–40

| Domains | Low risk | Probably low risk | Probably high risk | High risk | Not applicable | Comment | Quote (indicate reference) |
| --- | --- | --- | --- | --- | --- | --- | --- |
| 1. Study group |  |  | X |  |  | Probability sampling methods were employed. However, 30% of sampled households and 32% of sampled individuals were not interviewed. | The Americans changing lives (ACL) survey is a stratified, multistage area probability sample of noninstitutionalized civilian adults age 25 years and older living in the coterminous United States, with an oversampling of blacks and persons aged 60 years and older.’ The ACL wave 1 survey, conducted in 1986, interviewed 3617 persons face-to-face (representing 70% of sampled household and 68% of sampled individuals). |
| 1. Blinding |  | X |  |  |  | Absence of blinding judged as not significantly impacting outcomes. |  |
| 1. Exposure assessment |  | X |  |  |  | Working hours were self-reported, which has been proved to provide precise estimates. | Information retrieved from the study codebook. |
| 1. Outcome assessment |  |  | X |  |  | Instruments for prospective record of alcohol consumption were not applied, nor validated methods such AUDIT. Participants may have overestimated or underestimated alcohol use. | ‘Alcohol consumption was coded using 3 categories based on the number of drinks consumed in the past month: nondrinkers (0 drinks in the past month), moderate drinkers (1–89 drinks), and heavy drinkers (90 or more drinks).’ |
| 1. Confounding |  | X |  |  |  | Instruments for prospective record of alcohol consumption were not applied, nor validated methods such AUDIT. Participants may have overestimated or underestimated alcohol use. |  |
| 1. Incomplete outcome data |  | X |  |  |  | Attrition rate between 1986-1989 was 17%. | ‘The Americans’ Changing Lives (ACL) study is the oldest ongoing nationally representative longitudinal study of the role of a broad range of social, psychological, and behavioral factors (along with aspects of medical care and environmental exposure) in health and the way health changes with age over the adult life course. The study began in 1986 with a national face-to-face survey of 3617 adults ages 25 and up in the continental U.S., with African Americans and people aged 60 and over over-sampled at twice the rate of the others, and face-to-face re-interviews in 1989 of 83% (n=2867) of those still alive. Survivors have been re-interviewed by telephone, and where necessary face-to-face, in 1994, 2001/02, and 2011/12.’ |
| 1. Selective outcome reporting |  |  |  |  | X | Unpublished study |  |
| 1. Conflict of interest | X |  |  |  |  | Funding sources from governmental agencies. | ‘This study was supported by Grants P01AG05561 and R01AG09978-01 from the National Institute on Aging, National Institutes of Health, Bethesda, MD, and by a Health Investigator Award (to Dr. House) from the Robert Wood Johnson Foundation, Princeton, NJ.’ |
| 1. Other bias | X |  |  |  |  | No other source of bias was identified. |  |

# Study: ALAMEDA

Date of Assessment: October 7^th^ 2019

Life-Style and Future Health: Evidence from the Alameda County Study. Wiley JA, Camacho TC. Preventive Medicine 9, 1-21 (1980)

| Domains | Low risk | Probably low risk | Probably high risk | High risk | Not applicable | Comment | Quote (indicate reference) |
| --- | --- | --- | --- | --- | --- | --- | --- |
| 1. Study group | X |  |  |  |  | Probability sampling methods were employed. | ‘In 1965, the Human Population Laboratory conducted a sample survey of the noninstitutionalized adult residents of Alameda County, using mail-back questionnaires that were placed in sampled households by interviewers.’ |
| 1. Blinding |  | X |  |  |  | Absence of blinding judged as not significantly impacting outcomes. |  |
| 1. Exposure assessment |  | X |  |  |  | Working hours were self-reported, which has been proved to provide precise estimates. |  |
| 1. Outcome assessment |  |  | X |  |  | Instruments for prospective record of alcohol consumption were probably NOT applied, nor validated methods such AUDIT. Participants may have overestimated or underestimated alcohol use. |  |
| 1. Confounding |  | X |  |  |  | Analysis were adjusted to most important confounders (age, sex, SES), but other confounders may also be relevant. |  |
| 1. Incomplete outcome data |  |  | X |  |  | Attrition rate of 23,8%. |  |
| 1. Selective outcome reporting |  |  |  |  | X | Unpublished study | ‘In 1965, the Human Population Laboratory conducted a sample survey of the non-institutionalized adult residents of Alameda County, using mail-back questionnaires that were placed in sampled households by interviewers. Information about health status and life-style was obtained from 6,928 adults (persons over 20 or ever married) who were selected by area probability sampling methods. The sample used for this report consists of 5,108 white respondents who were less than 70 years of age in 1965.2 Of the 5,108 respondents, 76.2% (3,892) were located and successfully reinterviewed in a 1974 follow-up study (see Table 1).’ ‘The dropout group includes a higher proportion of males and of younger persons than does the panel group; they are also more apt to be in the lower income categories and to have fewer years of schooling. In addition, dropouts tend to engage in more high-risk behavior (e.g., smoking, heavy drinking) than do panel members. This is indicated by lower average scores in the dropout group on an index of health practices (described below). |
| 1. Conflict of interest |  | X |  |  |  | Funding sources were not reported in the retrieved study publications, but bias related to funding sources is unlikely. |  |
| 1. Other bias | X |  |  |  |  | No other source of bias was identified. |  |

# Study: BCS

Date of Assessment: October 7^th^ 2019

Childhood Mental Ability and Adult Alcohol Intake and Alcohol Problems: The 1970 British Cohort Study. Batty et AL. Am J Public Health. 2008; 98:2237–2243. doi:10.2105/AJPH.2007.109488.

Tarek Mostafa, Richard D Wiggins The impact of attrition and non-response in birth cohort studies: a need to incorporate missingness strategies Longitudinal and Life Course Studies 2015 Volume 6 Issue 2 Pp 131 – 146

| Domains | Low risk | Probably low risk | Probably high risk | High risk | Not applicable | Comment | Quote (indicate reference) |
| --- | --- | --- | --- | --- | --- | --- | --- |
| 1. Study group | X |  |  |  |  | Inclusion criterion was date or birth. | ‘The 1970 British Cohort Study (BCS70) is following the lives of around 17,000 people born in England, Scotland and Wales in a single week of 1970.’ |
| 1. Blinding |  | X |  |  |  | Absence of blinding judged as not significantly impacting outcomes. |  |
| 1. Exposure assessment |  | X |  |  |  | Working hours were self-reported, which has been proved to provide precise estimates. |  |
| 1. Outcome assessment |  |  | X |  |  | Instruments for prospective record of alcohol consumption were not applied, nor validated methods such AUDIT. Participants may have overestimated or underestimated alcohol use. | ‘Participants were asked how frequently they had an alcoholic drink of any kind. Participants who said that they currently drank were asked to provide details of their alcohol intake during the last 7 days.’ |
| 1. Confounding |  | X |  |  |  | Analysis were adjusted to most important confounders (age, sex, SES), but other confounders may also be relevant. |  |
| 1. Incomplete outcome data |  |  | X |  |  | 27 percent of attrition | ‘In table 1 we summarise the pattern of missing data for BCS70 over the nine waves of data collection from 1970 to 2012. Just under 1 in 5 (19.8 per cent, labelled as non-missing) of the CMs participated in all nine waves, whereas over half (52 per cent, labelled as non-monotone) dropped out from at least one wave but returned to the study in a subsequent wave, and nearly a third (27.2 per cent, labelled as monotone) dropped out from the survey after participating in a number of waves without ever returning, to date. The base sample of 17,284 CMs consists of the original birth sample (i.e. excluding immigrants who joined the study later on).’ |
| 1. Selective outcome reporting |  |  |  |  | X | Unpublished study |  |
| 1. Conflict of interest | X |  |  |  |  | No conflict of interest detected. | ‘BCS70 is core funded by the Economic and Social Research Council. The most recent sweep, at age 46, received additional funding from the Medical Research Council and and the British Heart Foundation.’ |
| 1. Other bias | X |  |  |  |  | No other source of bias was identified. |  |

# Study: MIDUS

Date of Assessment: October 7^th^ 2019

Spousal Problems and Family-to-Work Conflict Among Employed US Adults. Marshal Neal Fettro and Kei Nomaguchi. J Fam Econ Iss (2018) 39:277–296. DOI 10.1007/s10834-017-9555-2

Socioeconomic Status Moderates Genetic and Environmental Effects on the Amount of Alcohol Use. Nayla R. Hamdi, Robert F. Krueger, and Susan C. South. ALCOHOLISM: CLINICAL AND EXPERIMENTAL RESEARCH 2015; 39(4):603-610

<http://midus.wisc.edu/data/timeline.php>

| Domains | Low risk | Probably low risk | Probably high risk | High risk | Not applicable | Comment | Quote (indicate reference) |
| --- | --- | --- | --- | --- | --- | --- | --- |
| 1. Study group |  | X |  |  |  | National probabilistic sample. Analysis were adjusted for the profile of respondents. | ‘For this paper, of the 3343 respondents in the 2011 MIDUS-RS, we first selected 2476 respondents who were married to or cohabiting with a partner. Next, following prior research (Grzywacz and Marks 2000), we included those under the age of 62 only (n=1825). Then we selected those who were working for pay (n = 1434). Lastly, we restricted the sample to those who answered the SAQ, which resulted in the final sample of N=980. Using Heckman’s (1979) method, we evaluated possible bias from selecting respondents with a completed SAQ. Those included in our analytical sample were more likely to be older and have higher levels of education, and were less likely to be Hispanic. We then estimated the probability of being selected into the analytical sample (λ) and included it in our regression models. We found that λ had no significant effects in our models nor did it alter any patterns of findings discussed below, which suggests that our results were not biased by our sample restriction.’ |
| 1. Blinding |  | X |  |  |  | Absence of blinding judged as not significantly impacting outcomes. |  |
| 1. Exposure assessment |  | X |  |  |  | Working hours were self-reported, which has been proved to provide precise estimates. | ‘Respondent’s weekly work hours was measured as the number of hours of paid work at the respondent’s main job and any other jobs in a typical week’ |
| 1. Outcome assessment |  |  | X |  |  | No instrument for prospective record of alcohol consumption was applied, nor validated methods such AUDIT. Participants may have overestimated or underestimated alcohol use. | ‘Individuals were asked a number of survey questions related to alcohol use via mail-in self-administered questionnaire (MIDUS 2 main sample) or in-person survey (MIDUS Milwaukee sample). Participants reported age the first time he or she had a drink, the number of drinks currently consumed per week (or, if less than one per week, number of drinks per month), and how much a person was drinking at the time in their life when they were drinking the most. The questions regarding current drinking habits included: “During the last month, how often did you drink any alcoholic beverages, on the average? Would you say every day, 5 or 6 days a week, 3 or 4 days a week, 1 or 2 days a week, or less often than 1 day a week?” Individuals answering “less often than 1 day a week” would be further questioned if they drank “3 to 4 days per month, 1 to 2 days per month, or less often than that?” Participants were told to count as one 'drink', a bottle of beer, a wine cooler, a glass of wine, a shot of liquor, or a mixed drink. Participants were then asked: “On the days when you drank, about how many drinks did you drink, on the average?” The number of drinks per week was calculated by multiplying the answer to these two survey questions. ' |
| 1. Confounding |  | X |  |  |  | Analysis were adjusted to most important confounders (age, sex, SES), but other confounders may also be relevant. |  |
| 1. Incomplete outcome data |  |  | X |  |  | Attrition rate of at least 31% | MIDUS Data Timelines |
| 1. Selective outcome reporting |  |  |  |  | X | Unpublished study |  |
| 1. Conflict of interest | X |  |  |  |  | No conflict of interest detected. | ‘MacArthur Foundation Research Network on Successful Midlife Development’ |
| 1. Other bias | X |  |  |  |  | No other source of bias was identified. |  |

# Study: NSFH

Date of Assessment: October 7^th^ 2019

O’Leary and Schumacher. Addictive Behaviors 28 (2003) 1575 – 1585

Mirowsky and Reynolds, 2000. Sociological Methods and Research 28(4):476-504

| Domains | Low risk | Probably low risk | Probably high risk | High risk | Not applicable | Comment | Quote (indicate reference) |
| --- | --- | --- | --- | --- | --- | --- | --- |
| 1. Study group | X |  |  |  |  | National multistage probability sample | The main sample was a national multistage probability sample in which respondents participated on average in a 100-min interview. The target population consisted of English- or Spanish-speaking persons, 19 years and older, who lived in a household' |
| 1. Blinding |  | X |  |  |  | Absence of blinding judged as not significantly impacting outcomes. |  |
| 1. Exposure assessment |  | X |  |  |  | Working hours were self-reported, which has been proved to provide precise estimates. |  |
| 1. Outcome assessment |  |  | X |  |  | No instrument for prospective record of alcohol consumption was applied, nor validated methods such AUDIT. Participants may have overestimated or underestimated alcohol use. | In the NSFH, respondents were asked to report the ‘‘number of times drinking any alcohol in the last 30 days,’’ and their ‘‘average alcohol consumption when drinking in the last 30 days".' |
| 1. Confounding |  | X |  |  |  | Analysis were adjusted to most important confounders (age, sex, SES), but other confounders may also be relevant. |  |
| 1. Incomplete outcome data |  |  | X |  |  | Attrition rate of 23.9% | "The attrition rate of 23.9 percent seems high enough to substantial bias" |
| 1. Selective outcome reporting |  |  |  | X |  | Unpublished study |  |
| 1. Conflict of interest | X |  |  |  |  | No conflict of interest detected. | NSFH3 is being jointly funded by NICHD (Center for Population Research of the National Institute of Child Health and Human Development) grant # HD21009 and by NIA (National Institute on Aging), grant # AG10266. These two agencies also jointly supported NSFH2.' |
| 1. Other bias | X |  |  |  |  | No other source of bias was identified. |  |

**PRISMA checklist**

| **Section/topic** | **#** | **Checklist item** | **Reported on page #** |
| --- | --- | --- | --- |
| **TITLE** | | |  |
| Title | 1 | Identify the report as a systematic review, meta-analysis, or both. | 3 |
| **ABSTRACT** | | |  |
| Structured summary | 2 | Provide a structured summary including, as applicable: background; objectives; data sources; study eligibility criteria, participants, and interventions; study appraisal and synthesis methods; results; limitations; conclusions and implications of key findings; systematic review registration number. | 6-8 |
| **INTRODUCTION** | | |  |
| Rationale | 3 | Describe the rationale for the review in the context of what is already known. | 9-13 |
| Objectives | 4 | Provide an explicit statement of questions being addressed with reference to participants, interventions, comparisons, outcomes, and study design (PICOS). | 14 |
| **METHODS** | | |  |
| Protocol and registration | 5 | Indicate if a review protocol exists, if and where it can be accessed (e.g., Web address), and, if available, provide registration information including registration number. | 8 |
| Eligibility criteria | 6 | Specify study characteristics (e.g., PICOS, length of follow-up) and report characteristics (e.g., years considered, language, publication status) used as criteria for eligibility, giving rationale. | 14-17 |
| Information sources | 7 | Describe all information sources (e.g., databases with dates of coverage, contact with study authors to identify additional studies) in the search and date last searched. | 17-19 |
| Search | 8 | Present full electronic search strategy for at least one database, including any limits used, such that it could be repeated. | 69-77 (Appendix 2) |
| Study selection | 9 | State the process for selecting studies (i.e., screening, eligibility, included in systematic review, and, if applicable, included in the meta-analysis). | 19 |
| Data collection process | 10 | Describe method of data extraction from reports (e.g., piloted forms, independently, in duplicate) and any processes for obtaining and confirming data from investigators. | 19 |
| Data items | 11 | List and define all variables for which data were sought (e.g., PICOS, funding sources) and any assumptions and simplifications made. | 19-20 |
| Risk of bias in individual studies | 12 | Describe methods used for assessing risk of bias of individual studies (including specification of whether this was done at the study or outcome level), and how this information is to be used in any data synthesis. | 20-21 |
| Summary measures | 13 | State the principal summary measures (e.g., risk ratio, difference in means). | 21-23 |
| Synthesis of results | 14 | Describe the methods of handling data and combining results of studies, if done, including measures of consistency (e.g., I^2^) for each meta-analysis. | 23 |
| Risk of bias across studies | 15 | Specify any assessment of risk of bias that may affect the cumulative evidence (e.g., publication bias, selective reporting within studies). | 23 |
| Additional analyses | 16 | Describe methods of additional analyses (e.g., sensitivity or subgroup analyses, meta-regression), if done, indicating which were pre-specified. | 22-23 |
| **RESULTS** | | |  |
| Study selection | 17 | Give numbers of studies screened, assessed for eligibility, and included in the review, with reasons for exclusions at each stage, ideally with a flow diagram. | 26, 79-80 |
| Study characteristics | 18 | For each study, present characteristics for which data were extracted (e.g., study size, PICOS, follow-up period) and provide the citations. | 27-34 |
| Risk of bias within studies | 19 | Present data on risk of bias of each study and, if available, any outcome level assessment (see item 12). | 36-42, 81-97 |
| Results of individual studies | 20 | For all outcomes considered (benefits or harms), present, for each study: (a) simple summary data for each intervention group (b) effect estimates and confidence intervals, ideally with a forest plot. | 42-51 |
| Synthesis of results | 21 | Present results of each meta-analysis done, including confidence intervals and measures of consistency. | 42-51 |
| Risk of bias across studies | 22 | Present results of any assessment of risk of bias across studies (see Item 15). | 52-54 |
| Additional analysis | 23 | Give results of additional analyses, if done (e.g., sensitivity or subgroup analyses, meta-regression [see Item 16]). | 44-51 |
| **DISCUSSION** | | |  |
| Summary of evidence | 24 | Summarize the main findings including the strength of evidence for each main outcome; consider their relevance to key groups (e.g., healthcare providers, users, and policy makers). | 54-57 |
| Limitations | 25 | Discuss limitations at study and outcome level (e.g., risk of bias), and at review-level (e.g., incomplete retrieval of identified research, reporting bias). | 58 |
| Conclusions | 26 | Provide a general interpretation of the results in the context of other evidence, and implications for future research. | 59 |
| **FUNDING** | | |  |
| Funding | 27 | Describe sources of funding for the systematic review and other support (e.g., supply of data); role of funders for the systematic review. | 60 |
